# A Synchronous Strategy to Zn-Iodine Battery by Polycationic Long-Chain Molecules

**DOI:** 10.1007/s40820-025-01854-6

**Published:** 2025-07-17

**Authors:** Da-Qian Cai, Hengyue Xu, Tong Xue, Jin-Lin Yang, Hong Jin Fan

**Affiliations:** 1 Interdisciplinary Graduate Programme - Collaborative Initiative, Graduate College, Nanyang Technological University, Singapore, 637371 Singapore; 2 School of Physical and Mathematical Sciences, Nanyang Technological University, Singapore, 637371 Singapore; 3 Department of Chemistry, Tsinghua University, Beijing, 100084 People’s Republic of China; 4 School of Materials Science and Engineering, North Minzu University, Yinchuan, 750021 People’s Republic of China; 5 Energy Research Institute @ NTU (ERI@N), Nanyang Technological University, Singapore, 637553 Singapore

**Keywords:** Polyiodide shuttle effect, Halogen battery, Conversion cathode, Dendrites, Polycation molecule

## Abstract

Aqueous Zn-iodine batteries (ZIBs) face the formidable challenges towards practical implementation, including metal corrosion and rampant dendrite growth on the Zn anode side, and shuttle effect of polyiodide species from the cathode side. These challenges lead to poor cycle stability and severe self-discharge. From the fabrication and cost point of view, it is technologically more viable to deploy electrolyte engineering than electrode protection strategies. More importantly, a synchronous method for modulation of both cathode and anode is pivotal, which has been often neglected in prior studies. In this work, cationic poly(allylamine hydrochloride) (Pah^+^) is adopted as a low-cost dual-function electrolyte additive for ZIBs. We elaborate the synchronous effect by Pah^+^ in stabilizing Zn anode and immobilizing polyiodide anions. The fabricated Zn-iodine coin cell with Pah^+^ (ZnI_2_ loading: 25 mg cm^−2^) stably cycles 1000 times at 1 C, and a single-layered 3 × 4 cm^2^ pouch cell (N/P ratio ~ 1.5) with the same mass loading cycles over 300 times with insignificant capacity decay.

**Supplementary Information:**

The online version contains supplementary material available at 10.1007/s40820-025-01854-6.

## Highlights

- A long chain polycation (Pah⁺) is propos ed to simultaneously regulate Zn anode deposition , mitigate side reactions and stabilize iodine cathode chemistry.
- The iodophilic and low diffusivity nature of Pah enables effective polyiodide immobilization, suppressing the shuttle effect and ensuring a stable redox environment.
- The Zn iodine battery delivers high areal capacity (~4 mAh cm^−2^ at 1 C) and excellent durability, with 95% capacity retained over 1000 cycles.

**Supplementary Information:**

The online version contains supplementary material available at 10.1007/s40820-025-01854-6.

## Graphical abstract

**Figure:**
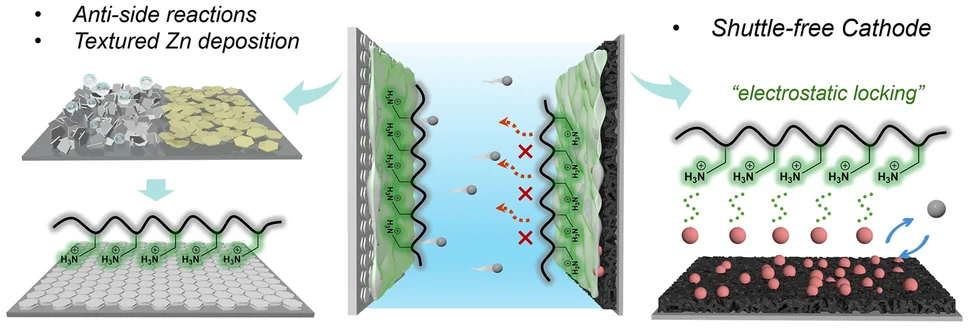

## Introduction

Rechargeable zinc ion batteries with intrinsic safety, eco-friendliness, and low-cost of both the Zn metal anode and aqueous electrolytes have raised great research interest [1]. Recently, aqueous zinc-iodine batteries (ZIBs) based on the redox couple of I^0^/I^−^ have emerged as an attractive setup for large-scale energy storage with relatively high specific capacity (211 mAh g^−1^) and abundant natural reserve (~ 55 μg L^−1^) in the ocean [2]. Despite these promising features, the technical issues on Zn metal anode and I_2_ cathode pose additional challenges to the practical implementation of ZIBs. On the anode side, the corrosion and passivation of Zn metal in mild acidic electrolytes jeopardize the Zn reversibility and deteriorate the performance. Moreover, the uncontrolled deposition of Zn results in dendrite formation, raising concerns on the short circuit of the battery [3]. On the cathode side, while favourable reaction kinetics can be achieved by the formation of highly soluble intermediates polyiodides (I_3_^−^ and I_5_^−^), it inevitably brings further issues of low coulombic efficiency and rapid deterioration in capacity upon cycling [2]. Worse still, the polyiodides diffused to the anode surface will corrode active Zn and acerbate battery degradation [2, 4]. Addressing the issues of low Zn reversibility, dendrite growth and polyiodide shuttling are pivotal to achieving practical Zn-iodine batteries.

Various strategies have been applied to tackle the issues related to Zn metal. Efforts on mitigating dendrite formation mainly focused on designing host or substrates for epitaxial Zn growth, as exemplified by graphene and vermiculite, with low lattice mismatch to hexagonal Zn crystal [5–7]. Constructing protective layers on Zn surfaces and modulating electrolyte compositions are widely adopted to shield Zn metal from active water, thereby alleviating water-related side reactions [8–11]. Notably, electrolyte modulation with high tunability and do not compromise manufacturing cost has raised wide attention recently [12–14]. For alleviating the shuttle effect of polyiodides, extensive progress has been made in exploiting hosts for iodine with physical/chemical confinement to polyiodides and catalytic conversion effect [15–17]. Notably, the incorporation of transition metal single-atom catalysts into iodine hosts has proven effective in enhancing iodine redox kinetics and utilization, thereby realizing zinc-iodine batteries with fast reaction rates and long-term cycling stability [18, 19]. Compared to the arduous preparation of hosts for iodine loading, regulating the electrolyte composition offers a more feasible and cost-effective approach to stabilize iodine cathode through multiple interactions [20–22]. The polyiodides with weak Lewis basicity can be chemically immobilized through spontaneous Lewis acid–base coordination and/or ion–dipole interactions [23, 24]. Considering the anionic property of polyiodides, cationic molecules were further proposed to capture polyiodides through strong Coulombic interaction [25]. Typically, quaternary ammonium salts were employed to trigger the formation of molecules-polyiodides complex, thereby mitigating the dissolution and shuttle of intermediate I_3_^−^ and I_5_^−^ [26–28]. Nevertheless, the formation of solid-state complex may sacrifice the fast reaction kinetics of polyiodides that benefit from the liquid reaction [29]. Therefore, exploring strategies to regulate the Zn metal anode and iodide cathode synergistically while maintaining the favourable kinetics behaviour of iodide species, is necessary for developing Zn-iodine batteries.

**Fig. 1:**
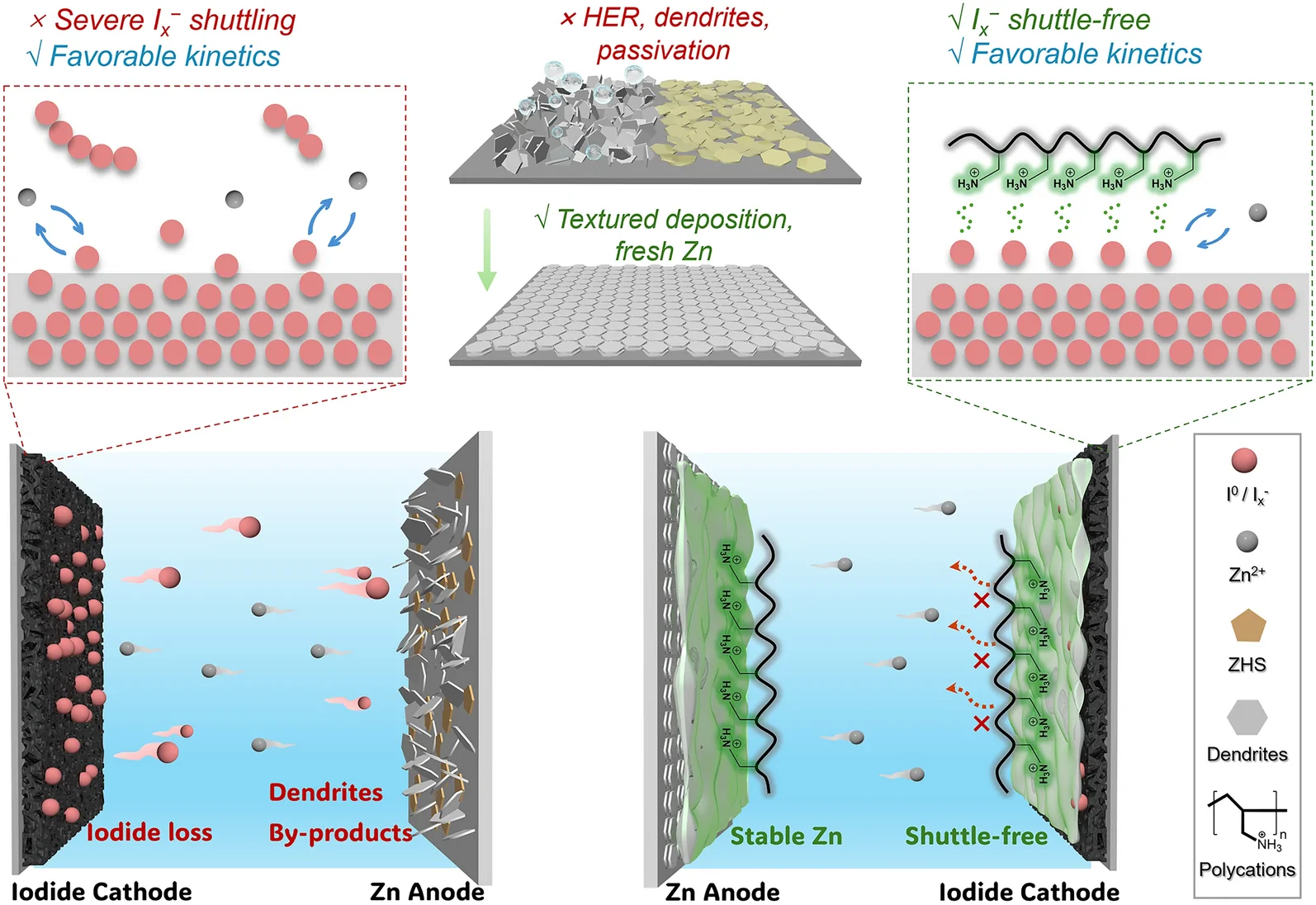
Schematics of the issues associated with aqueous Zn-iodine batteries and the regulation mechanism by cationic Pah^+^ long-chain molecules

In this work, we propose the synergistic optimization of the Zn metal anode and iodine cathode by dynamic adsorption of polycations at the electrode/electrolyte interfaces. The cationic poly allylamine hydrochloride (Pah^+^) with positive charge preferentially adsorb on the Zn anode and I_2_ cathode, as illustrated in Fig. 1. On the anode side, Pah^+^ adsorption reconstruct the electric double layer and interfacial electric field at Zn surface, contributing to homogeneous Zn deposition and suppressed side reactions. On the cathode side, Pah^+^ coordinates with anionic polyiodides to realize “electrostatic locking” effect due to Coulombic interaction. Because of the intrinsically low mobility of Pah^+^, the polyiodides shuttling is suppressed, accounting for the restricted self-diffusion. In addition to the coordination, the long-chain Pah^+^ also forms an adsorption layer on the cathode surface, spatially confining the iodine species from diffusing into the bulk electrolyte. As a result, we showcase the beneficial function of long-chain polycationic molecules as a synchronous strategy to mitigate issues in Zn-halogen batteries.

## Experimental Section

### Preparation of Electrolytes

ZnSO_4_·7H_2_O powder was first dissolved in deionized water to form pure 2 M ZnSO_4_ aqueous solution. Then, poly (allylamine hydrochloride) powder was added into the 2 M ZnSO_4_ solution with a various of concentration to form homogeneous solution (denoted as Pah^+^). For the control electrolyte, additional 0.145 M ZnCl_2_ powder was mixed along with the baseline solution (denoted as ZS). 0.05 M ZnI_2_ and 0.05 M I_2_ powders were dissolved in ZS or Pah^+^ electrolytes to prepared ZnI_x_-based electrolytes for polyiodide symmetric cell tests. 0.05 M ZnI_2_ powders were dissolved in ZS or Pah^+^ to prepare electrolytes for I_2_ deposition tests.

### Preparation of Iodide Cathodes

ZnI_2_ powder, activated carbon (AC), and polytetrafluoroethylene (PTFE) were mixed in a mass ratio of 6:4:0.5 and ground in an agate mortar. Isopropanol was added to form a dough-like mixture, which was then sheared and rolled into a free-standing film using a rolling machine (MSK-2150-H5, Shenzhen Kejing Star Technology Co., Ltd.). The film was dried at 50 °C under vacuum for 12 h to remove moisture and residual isopropanol, followed by punching into Φ = 12 mm discs for coin cells or rectangle pieces for pouch cells.

### Cell Assembly

All coin cells were assembled in CR2032 configuration. Zn||Zn symmetric was assembled using two identical Zn foils (Φ = 12 mm), 80 μL of electrolyte, and a glass fibre separator (Φ = 16 mm). Zn||Cu and Zn||Ti asymmetric cells were assembled in the same way, except Cu or Ti foils were used as the working electrode. Zn-iodide full cells were assembled with Zn foil anode, the iodide composite cathode, and 160 μL of electrolytes. For ZnI_x_-based symmetric cells, 30 μL of ZnI_x_ electrolyte was added to each of two identical activated carbon electrodes. For I_2_ deposition tests, 30 μL of 0.05 M ZnI_2_-anolyte was added to a carbon nanofibre film, and 30 μL of electrolytes was added to the Zn foil anode. After galvanostatic charging, the cells were disassembled, and the carbon nanofibre electrode was dried for scanning electron microscope analysis.

### Electrochemical Measurement

Galvanostatic charge–discharge tests were conducted using CT3002A (Wuhan Landt) and BTS4000 (Neware) battery testers. Cyclic voltammetry (CV), linear sweep voltammetry (LSV), and electrochemical impedance spectroscopy (EIS) measurements were carried out on CHI760e and Autolab PGSTAT 128N (Metrohm) electrochemical workstations. Linear sweep voltammetry (LSV) of Zn||Ti asymmetric cells was conducted from 0 to − 0.3 V at a scan rate of 1 mV s^−1^. Cyclic voltammetry (CV) was carried out over − 0.2 to 1 V at 1 mV s^−1^. Alternating current voltammetry (ACV) was performed from 1 to 0.2 V at a frequency of 5 Hz and an amplitude of 6 mV. For Zn-iodide full cells, CV was performed within 0.6 to 1.6 V. EIS of Zn||Zn symmetric cell was measured over a frequency range of 100 kHz to 100 mHz, and Zn-iodide cells from 100 kHz to 10 mHz, both with a perturbation amplitude of 8 mV. All EIS data were subjected to Kramers–Kronig validation to ensure a relative residual error below 1%. DRT analysis was performed using DRT Tools [30].

### Characterization Methods

Phase composition was analysed by X-ray diffraction (XRD) using a Bruker D8 Advance with Cu Kα radiation (λ = 0.15418 nm). Surface morphology and elemental distribution were observed with a ZEISS SUPRA^®^55 scanning electron microscope (SEM) equipped with energy dispersive spectroscopy (EDS). X-ray photoelectron spectroscopy (XPS, Shimadzu Kratos Axis Supra) with Al K*α* radiation was used to analyse valence states. Fourier transform infrared (FTIR) spectra were collected using a Bruker Tensor. Raman spectra were measured using a Horiba LabRAM HR Evolution with a 633 nm laser. ^1^H chemical shifts in the electrolytes were recorded by 400 MHz nuclear magnetic resonance (NMR, JEOL ECA400). UV–vis spectra were measured using a Shimadzu UV-1900 spectrophotometer. Pole figures of Zn substrates were collected using Rigaku SmartLab. Dynamic light scattering of electrolytes was analysed using Malvern Zetasizer Nano ZS. ATR-FTIR spectra were recorded on a PerkinElmer Frontier.

### Simulation Methods

Density functional theory (DFT) calculations were carried out using the Vienna Ab initio Simulation Package (VASP) with the projector augmented wave (PAW) method [31]. The exchange–correlation interactions were described using the Perdew–Burke–Ernzerhof (PBE) functional within the generalized gradient approximation (GGA) [32] with dispersion correction considered (DFT-D3) [33]. A cutoff energy of 500 eV was used for the plane-wave basis set. Geometry optimizations were performed using a 1 × 1 × 1 Γ-cantered k-point mesh. All slab models included ~ 15 Å of vacuum along the z-direction. Structures were fully relaxed until the total energy converged below 1 × 10^−6^ eV and residual forces were less than 0.02 eV Å^−1^. Quantum chemical calculations were performed using the ORCA 6.0 package. Geometry optimizations employed the B3LYP hybrid functional with D3 dispersion correction, using the def2-TZVP (− f) basis set and the def2/J auxiliary basis within the RIJCOSX approximation to accelerate integral evaluation. Molecular dynamics (MD) simulations were performed using the Desmond package. Initial electrolyte models were built with Packmol to solvate mixed solutes in a periodic simulation box, ensuring the desired component ratios and density [34]. Simulations were carried out at 300 K using a Nosé–Hoover thermostat and a Parrinello–Rahman barostat to maintain constant temperature and pressure, respectively. The OPLS-AA force field was used to describe interatomic interactions [35]. The system underwent energy minimization, followed by 100 ps of NVT and 100 ps of NPT equilibration. A 50 ns production run was then conducted to sample the configurational space.

## Results and Discussion

### Regulation of Zn Metal Anode

**Fig. 2:**
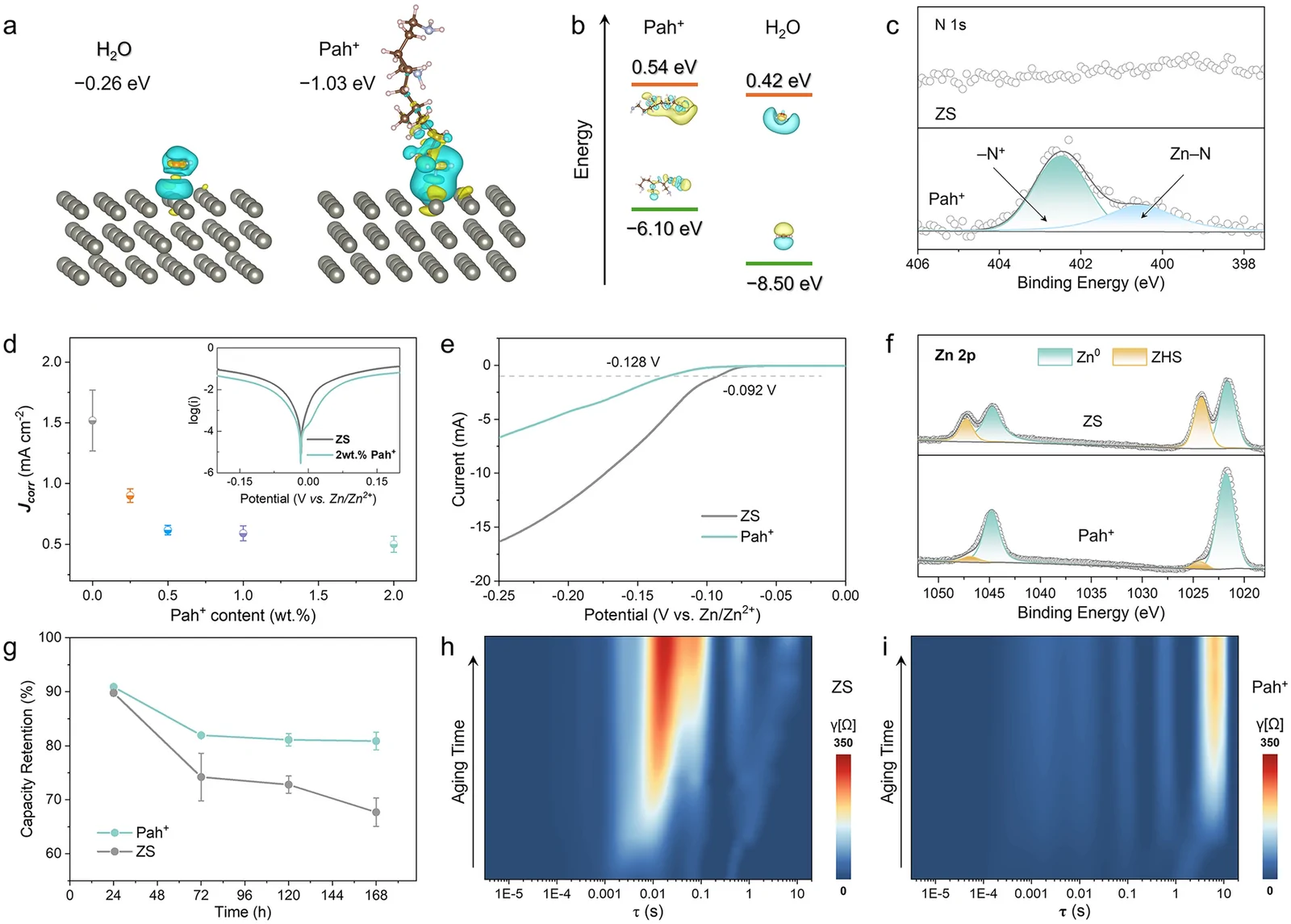
*Regulation of interfacial electrochemistry* **a** Adsorption and charge density difference of different molecules on Zn (002) surface, **b** HOMO/LUMO energy levels, **c** N 1*s* XPS spectra of Zn foils, **d** *J*_corr_ calculated from Tafel plots, **e** LSV curves, **f** Zn 2*p* XPS spectra, **g** Zn reversibility during the ageing process, and DRT plots of Zn||Zn cells in **h** ZS and **i** Pah^+^ during the ageing period of 12 h

Constructing a lean-water interface for Zn metal has been demonstrated an effective approach to alleviate interfacial side reactions for reversible Zn metal anodes. DFT calculation is employed to compare the interfacial adsorption affinity of different molecules (Fig. 2a). The calculated adsorption energy (E_ads_) of H_2_O molecules on Zn (002) is − 0.26 eV. In comparison, the Pah^+^ with the positively charged –N atoms bonded to Zn shows a more negative E_ads_ of − 1.03 eV. This indicates the more preferential adsorption of cationic Pah^+^ on Zn than H_2_O, as reflected by the electron accumulation at Pah^+^ molecule after adsorption. The calculated lowest unoccupied molecular orbital (LUMO) and the highest occupied molecular orbital (HOMO) (Fig. 2b) reveal that the Pah^+^ molecules possess a narrower energy gap compared to H_2_O. This narrower gap implies stronger electron transfer with Zn for effective adsorption.

The introduction of Pah^+^ in concomitant with Cl^−^ is realized by dissociation of poly allylamine hydrochloride. Considering the difference in anion polarity index between SO_4_^2−^ and Cl^−^ that may affect the Zn corrosion behaviours [36], equivalent amount of Cl^−^ was introduced in the control electrolyte (ZS). The surface adsorption of Pah^+^ was verified by XPS characterization of Zn foils immersed in the electrolytes. In N 1*s* spectra (Fig. 2c), a clear peak at 402.5 eV for quaternary ammonium group (–N^+^) is detected [37]. This is further verified by differential capacitance measured on alternating current voltammetry. The decreased capacitance in Pah^+^ implies the thickening of electric double layer due to competitive adsorption of Pah^+^ that expels hydrated ions and water molecules at the interface (Fig. S1), creating a lean-water interface [38]. Meanwhile, the other peak at lower binding energy of 400.5 eV reveals the interaction and electron transfer from Zn to –N^+^ in Pah^+^ that leads to the negative shift in binding energy [39]. The XPS depth profiles of Zn anodes after cycling were recorded to analyse the surface composition (Fig. S2). The N 1*s* signals detected on pristine Zn surface is attributed to the surface adsorption of Pah^+^, as previously discussed. However, the N 1*s* signals vanished after just 5 s of Ar^+^ sputtering. This suggests that Pah^+^ functioning as regulator of the Zn IHP structure does not decompose to form a solid-electrolyte interphase (SEI) layer, which aligns with the fact that Pah^+^ has a higher LUMO compared to H_2_O.

The hydrogen bond networks and Zn^2+^ solvation structure may be affected by the additional composition in the electrolyte, which gives rise to the regulated water decomposition behaviour [40]. In situ FTIR analysis of the Zn anodes surface during charging reveals a broad O–H stretching band within 2750–3700 cm^−1^ region (Fig. S3). In ZS, this band remains unchanged during the entire charging process. In comparison, the low-wavenumber shoulder of this band slightly intensifies as charging progresses in Pah^+^, indicating increased content of strongly hydrogen-bonded water molecules [41]. Raman spectrum of the electrolytes reveals lowered intensity at high shift region for Zn anode in Pah^+^ compared to ZS (Fig. S4), indicating a decrease in free H_2_O at the Zn surface. These spectral features imply the formation of a lean-water interface, likely resulting from the formation of strong hydrogen bonds between Pah⁺ and water adsorption that suppresses free water activity [42]. ^1^H NMR spectrum of ZS displays the signal with chemical shift at 4.70 (Fig. S5). With Pah^+^ addition, the chemical shift of ^1^H in H_2_O slightly upshift to 4.68, implying a decreased electron density around H nucleus which may be attributed to the reshaped coordination environment [43]. Molecular dynamics simulations were conducted to further check possible change in the Zn^2+^ solvation evolution (Fig. S6). In 2 M ZnSO_4_ electrolyte, the Zn^2+^ is surrounded by six water molecules, with a primary sharp peak at ~ 2 Å related to the Zn–O bonds in the hydrated Zn^2+^ in the radial distribution function (RDF). A new peak at 2.15 Å in RDF for the Pah^+^-containing electrolyte is ascribed to the Zn-N coordination. This indicates the unprotonated –NH_2_ groups containing lone-pair electrons may interaction with hydrated Zn^2+^ through Lewis acid–base interaction and thereby affect the solvation behaviour. The charge transfer resistance (R_ct_) of symmetric cells at varying temperatures were measured by EIS to assess the interfacial redox kinetics (Fig. S7). The calculated activation energy (E_a_) is lower in Pah^+^ (36 kJ mol^−1^) than in ZS (56 kJ mol^−1^). This might be correlated to the accelerated desolvation kinetics due to the interaction between the Zn^2+^ and –NH_2_ groups on the interfacial adsorbed molecules [44].

Due to the lean-water interface regulated by Pah^+^, the water-related interfacial side reactions (corrosion and HER) could be reduced. The corrosion current density (*J*_corr_) is calculated to be around 1.61 mA cm^−2^ in ZS (Fig. 2d). However, a trace amount of 0.5 wt% Pah^+^ decreases the *J*_corr_ to 0.62 mA cm^−2^. Further increasing the concentration does not evidently affect the anti-corrosion behaviour, suggesting that a relatively low concentration (e.g., 0.5 wt%) is adequate to reconstruct the Zn/electrolyte interface. However, to meet the requirement of surface adsorption at the iodide cathode side, the 2 wt% concentration has been selected for the following analysis (see more discussion in Supplementary Information). Linear sweep voltammetry (LSV) tests demonstrate lower HER activity at Zn surface adsorbed with Pah^+^, with the onset potential decreases by 36 mV (@1 mA cm^−2^) from the bare Zn metal (Fig. 2e). The anti-corrosion and suppressed HER properties endowed by the Pah^+^ is conducive to maintaining high Zn reversibility during ageing and cycling [45, 46]. Therefore, less zinc hydroxide sulphate (ZHS) byproducts are formed on the surface of Zn metal when it is soaked in the electrolyte with Pah^+^ for 7 days, as revealed by XRD characterization (Fig. S8). In Zn 2*p* XPS spectra (Fig. 2f), clear signals for ZHS appear at 1024.6 and 1047.8 eV for ZS, which are much less evident in Pah^+^. SEM image reveals distinct large-size flakes on Zn soaked in bare electrolyte, whereas a clean and smooth surface is characterized for Zn foil in Pah^+^ (Fig. S9). Therefore, the capacity retention test in Pah^+^ electrolyte shows an initial decrease after 72 h ageing but then remains relatively stable even after 168 h with 80.9% of initial capacity preserved (Fig. 2g). In the ZS, however, the capacity decreases rapidly after 72 h of ageing and continue to drop during subsequent ageing with only a retention of 67%. The self-corrosion of Zn in the electrolyte not only deteriorates the Zn reversibility and cause Zn loss but also generates a layer of byproducts at the surface.

EIS and corresponding distribution of relaxation time (DRT) analysis were conducted to evaluate the electrochemical processes at Zn surfaces with different EDL structures [47]. During the ageing process in ZS (Fig. S10), the Zn||Zn symmetric cell exhibits a continuous increase in the charge transfer resistance (R_ct_) and reached 1050 Ω after 12 h. As for the cell in Pah^+^, a more stable interfacial impedance is retained during the ageing process, with a low R_ct_ of ~ 34.5 Ω for fresh cell and ~ 170 Ω after 12 h. The DRT results provide deeper understanding of the electrochemical behaviour of Zn in different electrolytes (Fig. 2h, i) [48]. Six deconvoluted peaks are identified, corresponding to six characteristic electrochemical processes (Fig. S11). For the Zn electrodes aged in ZS electrolyte, the highest peak corresponds to the desolvation of Zn(H_2_O)_6_^2−^ with the time constant at ~ 10 ms, implying the sluggish desolvation kinetics at passivated Zn surface. However, the peak is much lower in Pah^+^ with the γ(*τ*) below 50, implying that the desolvation process is thermodynamically favourable. The peak for the mass transfer process increases obviously in Pah^+^, this is ascribable to the lowered ions transport kinetics due to the steric hindrance long-chain Pah^+^ molecules in the electrolyte [49]. Nevertheless, the ionic conductivity (σ) of the Pah^+^ electrolyte still reaches 55.4 mS cm^−1^ (Fig. S12).

**Fig. 3:**
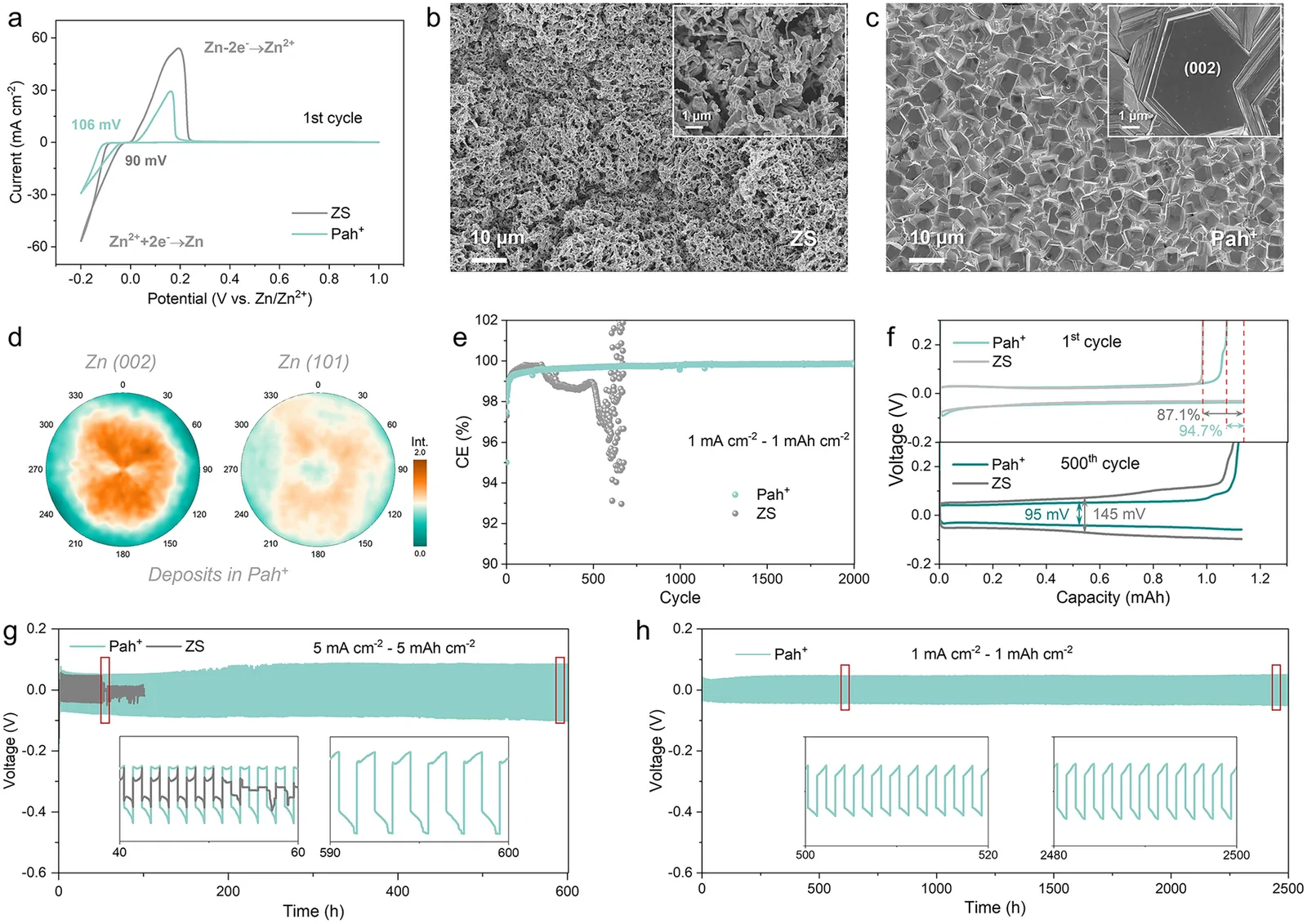
*Zn metal anode stability* **a** LSV of Zn||Ti cells, SEM images of Zn deposits in **b** ZS and **c** with Pah^+^, **d** XRD pole figures of Zn deposits in Pah^+^, **e** CE and **f** corresponding voltage profiles of Zn||Cu cells at selected cycles, long-term cycling tests of Zn||Zn cells at **g** 5 mA cm^−2^ for 5 mAh cm^−2^, and **h** 1 mA cm^−2^ for 1 mAh cm^−2^

The Zn^2+^ electrochemistry at Zn electrodes with different EDL structures was investigated by LSV test of Zn||Ti asymmetric cells (Fig. 3a). Typical redox peaks are observed for the cell using Pah^+^ electrolyte, corresponding to the Zn^2+^/Zn redox behaviour. The decrease in the enclosed peak area implies the lowered reactivity of Zn^2+^ at the Pah^+^-regulated interface. The elevated nucleation overpotential (η_n_) to 106 mV compared to the 90 mV in ZS indicates the hindered nucleation process with the presence of Pah^+^ at Zn/electrolyte interface. Similar trend is observed in Zn||Cu cells during galvanostatic discharging (Fig. S13). A higher η_n_ of 99 mV is detected in Pah^+^ than the 88 mV in ZS, with comparable η_g_ that resulted in higher ΔE of 70.3 mV than 61.0 mV in ZS. Though excessive driven force is required for the nucleation process, the higher η_n_ could benefit the deposition process by inducing finer critical nuclei during galvanostatic charging of Zn anode [50, 51]. These results are consistent with that of chronoamperometry (CA) tests (Fig. S14). With the fix bias potential of − 0.15 V, the period of current drop is much longer in ZS. In comparison, the current response becomes stable shortly after the decline at the initial stage for around 15 s in Pah^+^. This indicates that the 2D diffusion of Zn^2+^ at the anode surface has been restricted by interfacial adsorption of Pah^+^, contributing to more homogeneous ion flux to realize even deposition and less Zn dendrite formation [52]. The transference number of Zn^2+^ (t_Zn2+_) measured using the Bruce-Vicent method is around 0.52 in ZS (Fig. S15). The t_Zn2+_ increases to 0.73 with the existence of Pah^+^, which may contribute to the decreased anion concentration gradient at anode surface and suppressed formation of byproducts [53].

The surface roughness of Zn deposits was first characterized by confocal laser scanning microscopy (CLSM) at a broad scale (Fig. S16). The surface is rather uneven in ZS with a higher arithmetical mean height (S_a_) of 4.92 μm than that in Pah^+^(1.9 μm). Compared to the loose and porous Zn deposits in ZS, the Zn deposits are more compact and uniform on the anode charging in Pah^+^ under same testing condition of 2 mA cm^–2^ for 10 mAh cm^–2^ (Fig. 3b, c). Notably, a dense hexagonal texture is observed. The difference in crystallographic orientation of Zn deposits can be seen also in the XRD pole figure patterns, where (101) plane dominates for deposits in ZS (Fig. S17) whereas (002) for Zn deposit in Pah^+^ (Fig. 3d). This is consistent with the results of reshaped dominated diffraction planes in XRD (Fig. S18). DFT calculations show that Pah⁺ adsorption is more favourable on the (100) and (101) facets, with E_ads_ values of − 1.33 and − 1.26 eV, respectively, compared to –1.03 eV on the (002) facet (Fig. S19). As Pah^+^ adsorption inhibits both water-related side reactions and Zn^2+^ reduction kinetics, its stronger binding to the (100) and (101) facets is expected to suppress Zn growth along these directions, thereby promoting [0001] growth and preferential exposure of the (002) facet [54]. Ex situ XRD patterns of Zn anodes at various deposition times reveal the crystallographic evolution of Zn deposits (Fig. S20). The intensity ratio between (002) and (101) peak increases continuously throughout the plating process in Pah^+^ whereas the ratio shows negligible increase in ZS. Hence, the formation of (002) texture and preferentially exposed (002) facet may have contributed to improved Zn reversibility and absence of dendrites formation. After cycling at 1 mA cm^–2^ and 1 mAh cm^–2^, the Zn anode in Pah^+^ remains a flat surface without obvious aggregates and flakes. In sharp contrast, numerous fluffy flakes are formed on the Zn surface in ZS (Fig. S21). In situ optical microscopy offers a direct visualization of Zn deposition (Fig. S22). In the ZS electrolyte, the Zn foil develops random surface protrusions that gradually evolve into irregular deposits, whereas the Zn foil in the Pah^+^ electrolyte retains a smooth surface, suggesting that Pah^+^ adsorption effectively guides uniform Zn deposition.

Asymmetric Zn||Cu cells are assembled to evaluate the coulombic efficiency (CE) in different electrolytes (Fig. 3e). Drastic fluctuation in CE is observed for the cell in ZS after only 200 cycles and the cell fails in 670 cycles, with an average CE of 98.08%. For the cell with Pah^+^, the CE remains stable for 2000 cycles with higher average CE reaching 99.75% (initial CE is 94.7%) (Fig. 3f). The voltage profiles demonstrate gradual increase in voltage separation in ZS due to the accumulation of ZHS that impedes the Zn^2+^ reactivity, reaching 145 mV in 500 cycles. The voltage profiles remain more stable in Pah^+^, exhibiting a low voltage separation of 95 mV after 500 cycles, which indicates enhanced reversibility (Fig. S23). The rate capability of Zn electrodes is assessed in Zn||Zn symmetric cells. With gradually increasing current density, Pah^+^ demonstrates stable voltage profiles with slightly higher polarization than ZS (Fig. S24). During the long-term cycling test at 5 mA cm^−2^ for 5 mAh cm^−2^, the cell in ZS undergoes short circuit after only 55 h (Fig. 3g). The cell with Pah^+^ exhibits a longer (600 h) cycle life. Increasing the Pah^+^ concentration to 4 wt% does not increase the voltage hysteresis (Fig. S25). However, the stability of the symmetric cell deteriorates, probably due to the excessive amount of Cl^−^ [55]. During the long-term cycling test at 1 mA cm^−2^ for 1 mAh cm^−2^, the symmetric cell operates for over 2500 h with stable voltage hysteresis (Fig. 3h), while the cell in ZS becomes short circuit in less than 300 h (Fig. S26). The extended lifespan achieved with a low concentration of Pah^+^ is comparable to, or surpasses, that of recently reported additive systems (Table S1), demonstrating the effectiveness of this long-chain cationic molecule in stabilizing Zn metal.

### Inhibition of Polyiodides Shuttling

**Fig. 4:**
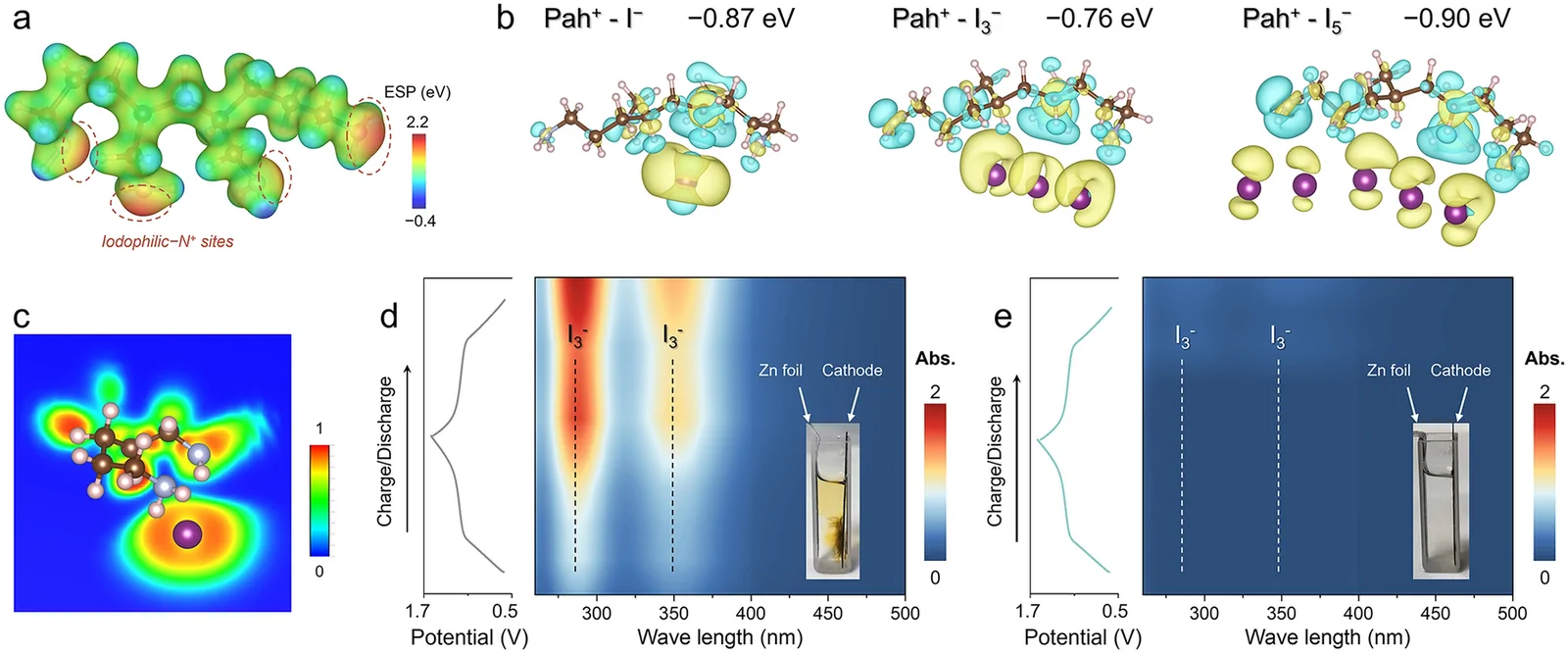
*Reduced shuttling of polyiodides by Pah*^+^ **a** Electrostatic potential mapping of Pah^+^, **b** charge density difference of adsorption configuration to I_x_^−^, **c** electron localization function (ELF) mapping of coordinated between Pah^+^ and I^−^, and **d, e** in situ UV–Vis spectra for I_3_^−^ absorbance in ZS and Pah^+^, respectively. Insets are the photographs of Zn-iodine batteries in quartz cell

Immobilizing the anionic I^−^/I_x_^−^ species through Coulombic interaction with cationic sites represents an effective strategy achieving shuttling-free Zn-I_2_ batteries [25]. The calculated ESP mapping for Pah^+^ units in Fig. 4a exhibit positively charged at –N^+^ sites, showing a good potential for polyiodides immobilization. Therefore, the adsorption energy of Pah^+^ to iodine species were calculated to identify the capability of polyiodide adsorption. As summarized in Fig. 4b, the adsorption energies are − 0.87, − 0.76, and − 0.90 eV for I^−^, I_3_^−^ and I_5_^−^, respectively, implying the chemisorption of iodide species by Pah^+^. According to the charge density difference of the optimized adsorption configuration, the electrons of negatively charged I_x_^−^ deviate from I sites and accumulate nearby Pah^+^. The 2D Electron Localization Function (ELF) map of charge density difference for Pah^+^ and I^−^ reveals that the electrons in I^−^ deviate to N sites in Pah^+^, illustrating the interaction between cationic Pah^+^ and polyiodide anions (Fig. 4c). To verify this, in situ UV–vis tests were conducted on Zn-iodine full batteries with different electrolytes using a homemade commercial quartz cell. ZnI_2_ as the initial active material loaded on activated carbon cathode was matched with Zn foil anode and different electrolytes. During the charging/discharging process, the absorbance of I_3_^−^ signals at ~ 287 and 351 nm wavelength gradually increases in ZS (Fig. 4d) [56]. After a complete charging and discharging process, the electrolyte turns yellow in the quartz cell, indicating the shuttling of polyiodides from cathode surface into the bulk electrolyte. In contrast, the absorbance of I_3_^−^ signals is much lower in Pah^+^ and the electrolyte remains colourless and transparent after the charging/discharging process (Fig. 4e). The EDS mapping of the cathode cross section after charging clearly show N signal due to Pah^+^ adsorption (Fig. S27), which indicates effective immobilization of iodine species by the long-chain cationic Pah^+^.

### Electrochemical Property of Zn-iodine Battery

**Fig. 5:**
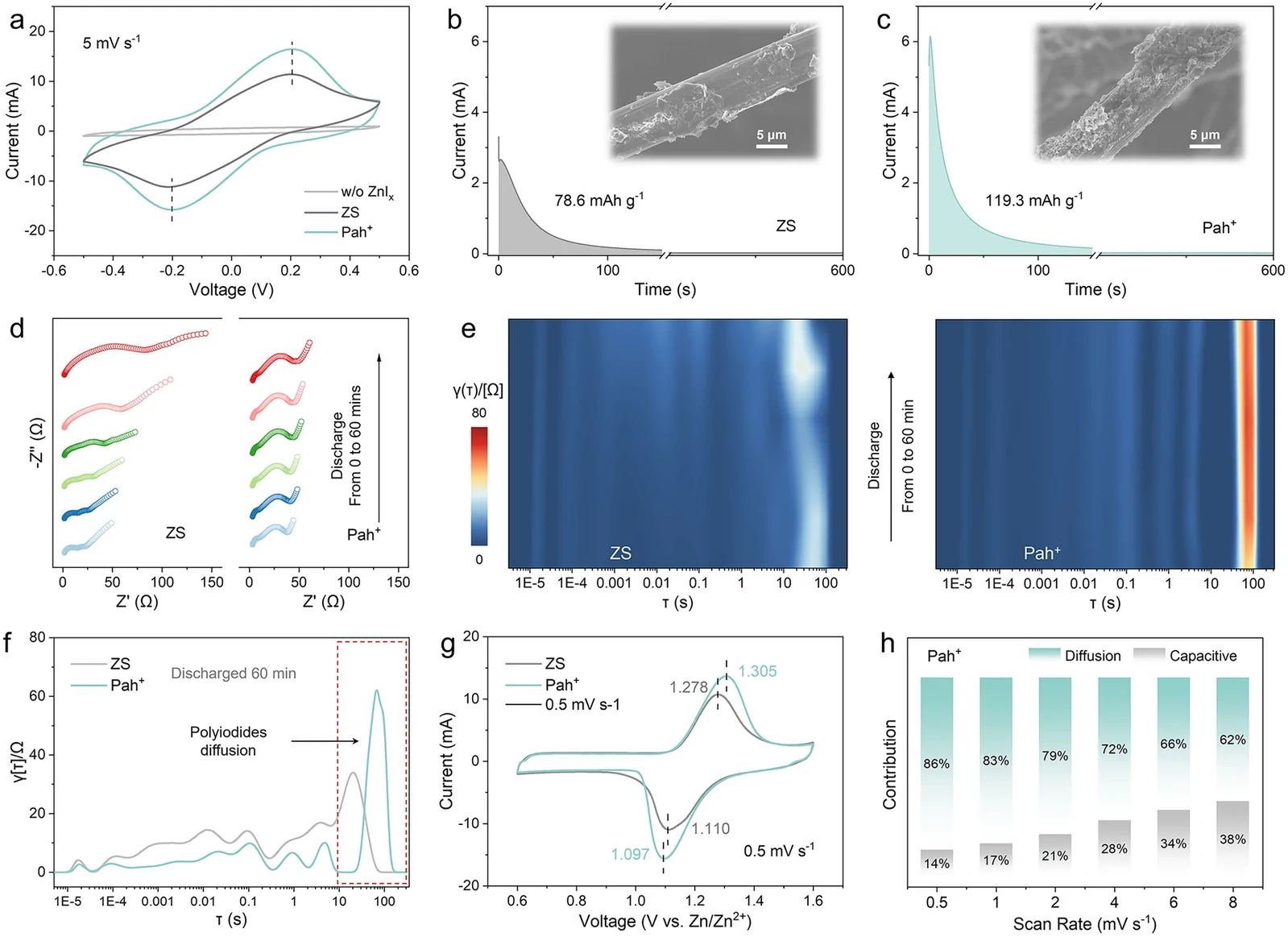
*Redox behaviour of Zn-iodine batteries* **a** CV of ZnI_x_-based symmetric cells, potentiostatic charge curves of 0.05 M ZnI_2_ in **b** ZS and **c** Pah^+^, inserted with SEM images of I_2_ deposition morphology. **d** EIS Nyquist plot of Zn-iodine full cell during discharging and **e** corresponding DRT plots in ZS and Pah^+^, **f** selected DRT plots after discharged for 60 min. **g** CV of Zn-iodine batteries, **h** contributions for the capacity at different scan rates

CV of symmetric cells with ZnI_x_-based electrolytes were conducted (Fig. 5a) to investigate the reversibility of iodine species. The long-chain Pah^+^ enables more efficient active material utilization, giving rise to a higher current response than the cell in ZS [15, 28]. The peak separation remains the same to ZS, indicating the redox kinetics has not been deteriorated by the soluble Pah^+^ molecules. I_2_ deposition behaviour was investigated by potentiostatic charging at 1.25 V (Fig. 5b, c) with the catholyte contain ZnI_2_. While both cells reach the max current at around 2 s, the current response is much higher in Pah^+^ than in ZS. The calculated I_2_ deposition capacity is higher in Pah^+^ (reaching 119.3 mAh g^−1^) than in ZS (78.6 mAh g^−1^). The SEM image reveals few rough deposited particles on carbon paper in ZS, while thick deposits are observed in Pah^+^. These results indicate the more efficient utilization of ZnI_2_ and polyiodide species with Pah^+^ that contribute to higher I_2_ deposition capacity [57].

To better understand the immobilization mechanism of polyiodides by Pah^+^, EIS and corresponding DRT analysis were conducted on Zn-iodine coin cells. The cells were first charged to 1.6 V to fully convert I^−^ of initial ZnI_2_ into I^0^. At the initial stage of discharge process, the cell in ZS shows a lower R_ct_ than in Pah^+^, probably ascribed to the fresh Zn surfaces endowing better reactivity compared to Pah^+^ adsorbed Zn surfaces (Figs. 5d and S28). However, as the discharge process progresses, the cell in ZS exhibits an obvious increase in R_ct_, while the cell in Pah^+^ retains a stable R_ct_. The DRT analysis provides deeper insights into the kinetics of each electrochemical process (Fig. 5e) [58]. Apart from the six typical peaks for the electrochemical processes identified in Zn||Zn symmetric cells, another deconvoluted peak appears for Zn-iodine full cells with a characteristic time constant in 10 ~ 100 s [8]. This is attributed to the mass transfer of iodide species in the electrolyte. Taking the cell discharged for 60 min at 1 mA cm^−2^ as a case study, the *γ*[*τ*] for each electrochemical process below ~ 10 s time constant is higher in ZS than Pah^+^, indicating the passivation of Zn anode that deteriorates the full cell performance (Fig. 5f). More importantly, the *γ*[*τ*] for iodide species diffusion further increases to over 60 by Pah^+^, which implies that the self-diffusion process of iodides becomes thermodynamically less favourable. Meanwhile, the time constant for this process exhibits an obvious shift to ~ 66 s in Pah^+^ than ~ 20 s in ZS. After the discharge process, the glass fibre membrane of the cell in ZS turns yellow, while it maintains colourless in Pah^+^ (Fig. S29). Dynamic light scattering (DLS) analysis shows a high diffusion coefficient exceeding 100 μm^2^ s^−1^ for ZS, corresponding to a smaller hydrodynamic diameter in the blank ZS electrolyte (Fig. S30) [59]. In contrast, in the presence of long-chain Pah^+^, a negative shift in diffusion distribution is observed, suggesting increased hydrodynamic diameters and a lower diffusion coefficient. Hence, we may infer that the polyiodides captured by Pah^+^ will not readily self-diffuse in the electrolyte.

To check if the diffusion-limited battery behaviour has been changed by the Pah^+^ surface adsorption, kinetic analysis was conducted from CV tests at various scan rates (Fig. S31). The CV profile at 0.5 mV s^−1^ in Fig. 5g show a pair of typical redox peaks for the I^0^/I^−^ conversion, demonstrating the unchanged reaction mechanism in Pah^+^. The polarization only slightly increases in Pah^+^ (40 mV in the voltage separation). However, the cell in Pah^+^ displays higher current responses and enclosed peak areas in CV tests at all scan rates than in ZS. This reflects a reduced shuttle effect of polyiodides and improved iodine utilization upon Pah^+^ adsorption. The ion storage behaviours in Zn-iodide batteries are further identified by fitting the peak current *i* and the scan rate *v* into the equation: $\mathrm{log}\left(i\right)=b\mathrm{log}\left(v\right)+\mathrm{log}(a)$. As seen in Fig. S31, the *b* values are both around 0.65, indicating the charge storage behaviour is dominated by diffusion process [60]. The quantitative analysis reveals that 86% of the capacity is contributed by the diffusion process in Pah^+^ (Fig. 5h). Further increasing the scan rate to 8 mV s^−1^, diffusion process still accounts for 62% of the capacity, implying that the contribution of I^0^/I^−^ redox pair dominates over the capacitance contributed by carbon materials in both ZS and Pah^+^ (Fig. S32).

### Zn-iodine Full Cells

**Fig. 6:**
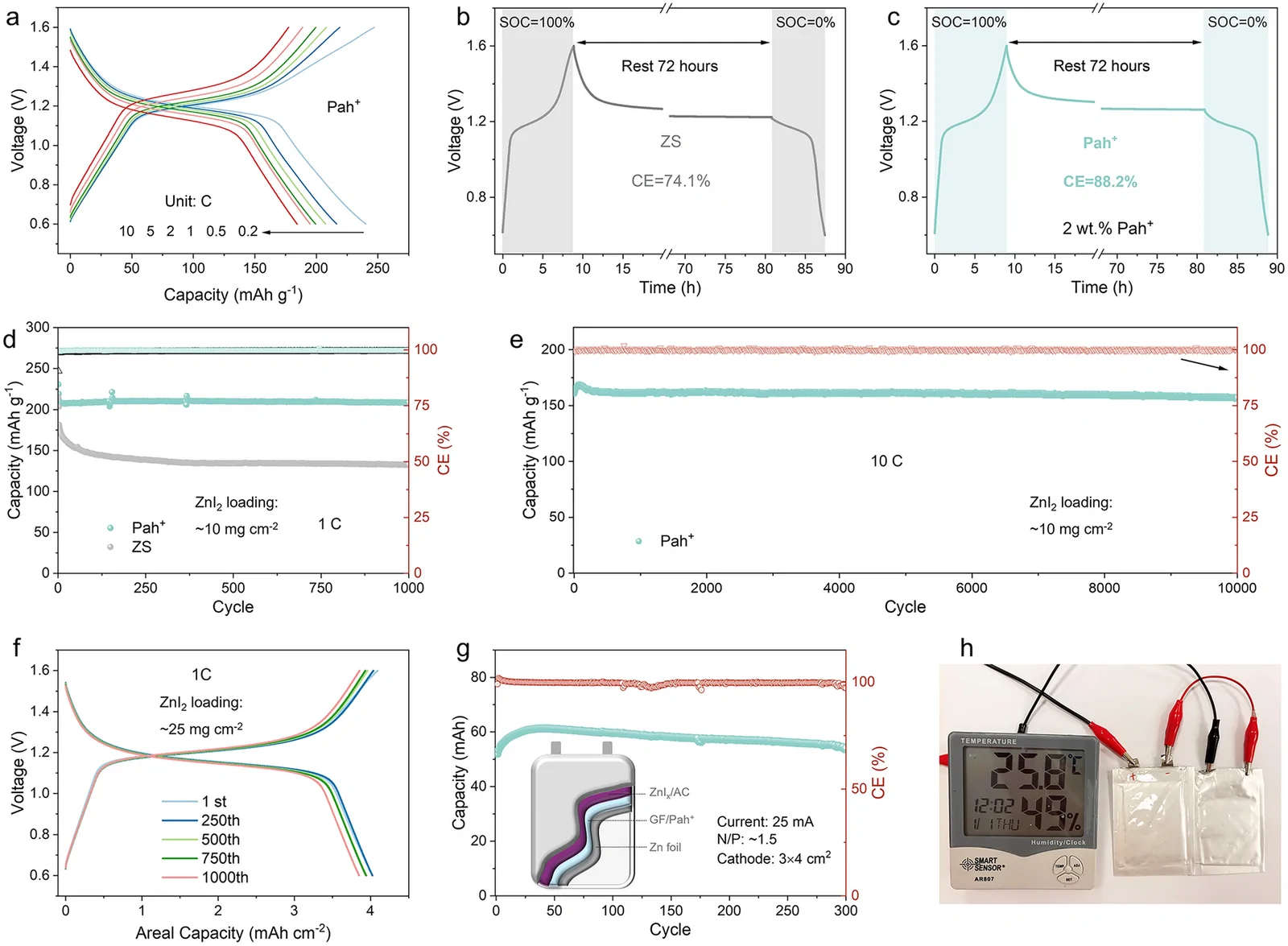
Electrochemical performance of Zn-iodine full cells. **a** Rate capability, **b** self-discharge behaviour of Zn-iodine batteries in **b** ZS and **c** Pah^+^, long-term cycling at **d** 1 C and **e** 10 C. **f** GCD curves of batteries with high ZnI_2_ loading, **g** pouch cell performance, and **h** digital photograph of pouch cells powering the electronic device

Zn-iodine full cells are fabricated with ZnI_2_ loading of ~ 10 mg cm^−2^. The cell in Pah^+^ delivers capacities of 240, 217, 208, 199, 195, and 185 mAh g^−1^ at the current density of 0.2, 0.5, 1, 2, 5, and 10 C (1 C = 211 mA g^−1^), respectively (Fig. 6a). The capacities in ZS are consistently lower at all current densities, which may stem from the polyiodide shuttling that leads to insufficient iodine utilization (Fig. S33). The dissolution and shuttle effect of polyiodides inevitably lead to self-discharge of full cell [61]. The cell in ZS retains only 74.1% capacity of the fully charged capacity after 72 h resting, showing an obvious self-discharge behaviour (Fig. 6b). Benefiting from the inhibited shuttle effect of polyiodides by Pah^+^, the capacity retention is improved to 88.2% (Fig. 6c). To better illustrate the effect of Pah^+^ in iodine cathode stabilization, we also tested neutral polyacrylamide (PAAm) additive (Fig. S34). While long-chain PAAm is effective in stabilizing the Zn anode as reported previously [62], its neutral structure lacks cationic sites for polyiodide immobilization via Coulombic interaction. That may explain the much lower capacity retention (77.8%) than that in Pah^+^ after 72 h rest. This highlights the critical role of dual-electrode stabilization provided by long-chain cationic molecules in Zn-iodine batteries.

Upon long-term cycling at 1 C, the full cell in Pah^+^ shows an initial capacity of 230 mAh g^−1^ at the first cycle; and a high capacity of 209 mAh g^−1^ is retained after 1000 cycles (Fig. 6d). In contrast, while the cell in ZS delivers a comparable capacity of 214 mAh g^−1^ at the initial cycle, the value rapidly drops to 153 mAh g^−1^ after 50 cycles due to severe shuttle effect (Fig. S35). Even at a high rate of 10 C, an initial capacity of around 170 mAh g^−1^ can be achieved with distinct charge/discharge plateaus (Figs. 6e and S36). After 10,000 cycles, nearly 93% of initial capacity is maintained, with a low decay of 0.0071 ‰ per cycle. When further increasing the mass loading of ZnI_2_ to 25 mg cm^−2^ (Fig. 6f), the coin cell delivers a specific capacity of nearly 200 mAh g^−1^, reaching an areal capacity of 4.0 mAh cm^−2^. The value slightly drops to 3.8 mAh cm^−2^ after 1000 cycles with an average CE of 99.7% (Fig. S37). Single-layer pouch cells are fabricated to evaluate the effectiveness for practical applications. A 3 × 4 cm^2^ cathode reaches a capacity of around 61 mAh in pouch cell with a low N/P ratio of around 1.5 at the current of 25 mA and can stably cycle over 300 times without obvious capacity decay (Figs. 6g and S38). Scaling up the cathode to 5 × 6 cm^2^ increases the capacity to 116 mAh at the same current density with stable cycle performance. Two pouch cells with 3 × 4 cm^2^ cathodes connected in series give an open circuit potential of 2.9 V (Fig. S39) which is sufficient to continuously power a thermo-hygrometer (Fig. 6h).

## Conclusion

The long-chain cationic molecule (Pah^+^) is proven an effective dual regulator for both Zn metal anode and iodine cathode in aqueous ZIBs. On the anode side, the positively charged − N^+^ sites and saturated alkyl groups of Pah^+^ endow the Zn surface with high hydrophobicity, contributing to the corrosion resistance (by both H_2_O and Cl^−^). The increased nucleation overpotential and restricted lateral Zn^2+^ diffusion account for the uniform Zn deposition without obvious dendrite formation. On the cathode side, the cationic Pah^+^ coordinates with I_x_^−^ anions through strong Coulombic interaction. The suppressed polyiodide shuttle effect is attributed to the inherently low diffusivity and interfacial confinement of iodine species by the cationic polymer adsorption layer. This effect, combined with the preserved liquid-phase polyiodide cathode (i.e., no precipitation), could have accounted for the low self-discharge behaviour without compromising iodine redox kinetics. The above merits ensure the efficacy of low additive concentration at high iodine loadings (25 mg cm^−2^ ZnI_2_).

We found no obvious change to the Zn solvation structure due to the absence of nucleophilic sites on the molecule chain, nor to the diffusion-dominated redox reaction of the full battery. In addition, the Pah^+^ molecule does not decompose to form a SEI on Zn metal surface, as implied by XPS depth profile analysis on Zn anode after cycling. This is consistent with the higher LUMO of Pah^+^ than H_2_O, which means the former is unlikely to be reduced to form SEI.

This work not only presents a facile and viable electrolyte engineering approach but also provides insights to the mechanism of synchronous modulation by the long-chain cationic molecule in Zn-iodine battery. However, the discussed beneficial effect is more significant at low current rates than high rates. The saturated Pah^+^ lacks zincophilic sites. Hence, while it suppresses the interfacial reactivity of water, it also slows down the Zn/Zn^2+^ redox kinetics, leading to an increase in polarization at the anode. A systematic exploration of polycationic molecules with multiple functional groups and their impact on the rate capability of Zn anodes should be done in future.

## Supplementary Information

Below is the link to the electronic supplementary material.

Supplementary file1 (DOCX 26779 KB)
