# Supplementary material for: A Synchronous Strategy to Zn-Iodine Battery by Polycationic Long-Chain Molecules
Source: Nanomicro Lett. 2025 Jul 17;18:3. doi: 10.1007/s40820-025-01854-6 (PMC12271045; doi:10.1007/s40820-025-01854-6)
Supplement: Supplementary file 1 — Supplementary file1 (DOCX 26779 KB) [file 40820_2025_1854_MOESM1_ESM.docx]

Supporting Information for

**A Synchronous Strategy to Zn-Iodine Battery by Polycationic Long-Chain Molecules**

Da-Qian Cai^1,2^, Hengyue Xu^3^, Tong Xue^4^, Jin-Lin Yang^2,5^, Hong Jin Fan^2,5,^ *

^1^ Interdisciplinary Graduate Programme - Collaborative Initiative, Graduate College, Nanyang Technological University, Singapore 637371, Singapore

^2^ School of Physical and Mathematical Sciences, Nanyang Technological University, Singapore 637371, Singapore

^3^ Department of Chemistry, Tsinghua University, Beijing 100084, P. R. China

^4^ School of Materials Science and Engineering, North Minzu University, Yinchuan 750021, P. R. China

^5^ Energy Research Institute @ NTU (ERI@N), Nanyang Technological University, Singapore 637553, Singapore

*Corresponding author. E-mail: [fanhj@ntu.edu.sg](mailto:fanhj@ntu.edu.sg) (Hong Jin Fan)

# **Supplementary Data**


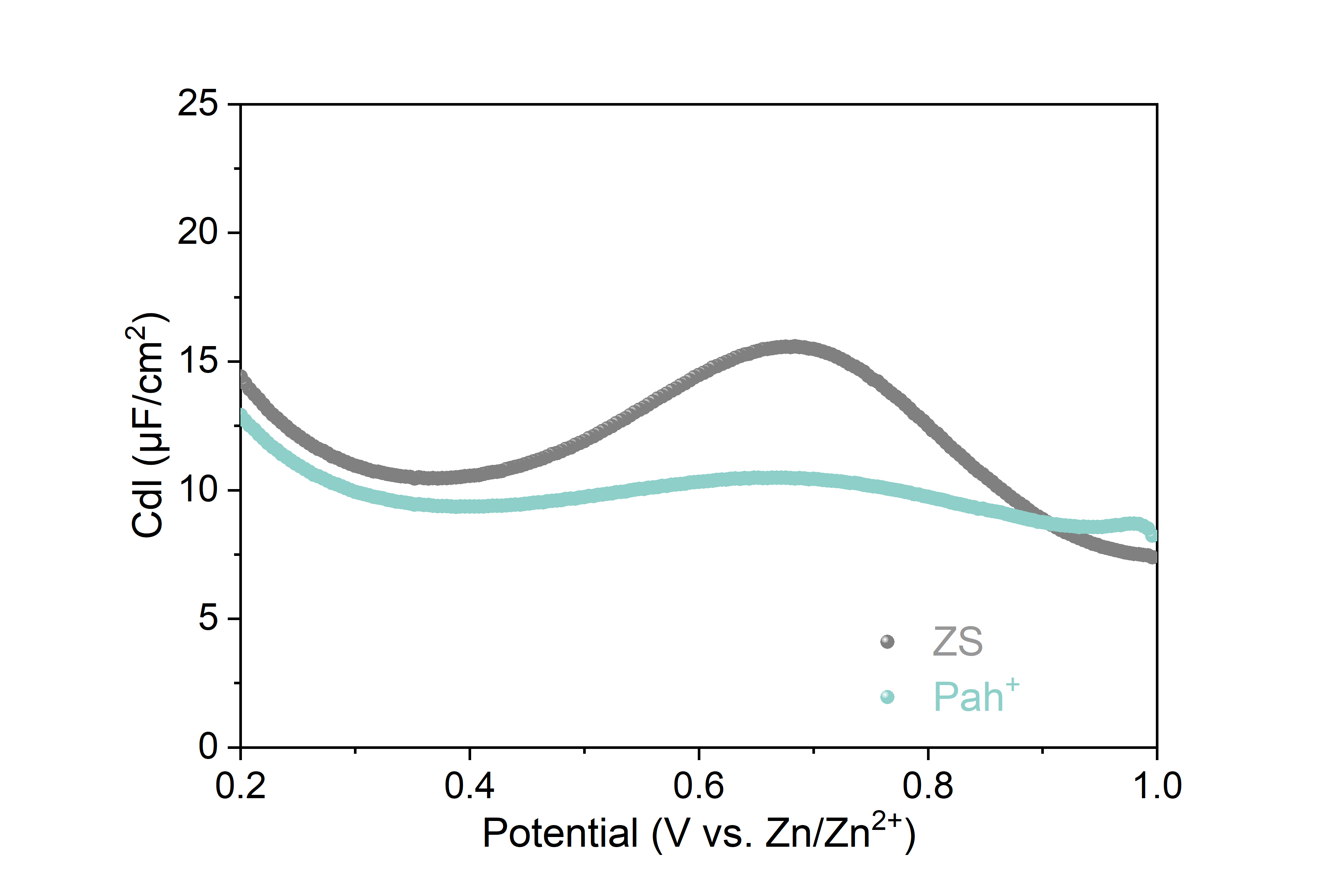


**Fig. S1** Differential capacitance-potential curves of Zn||Ti asymmetric cells measured by ACV


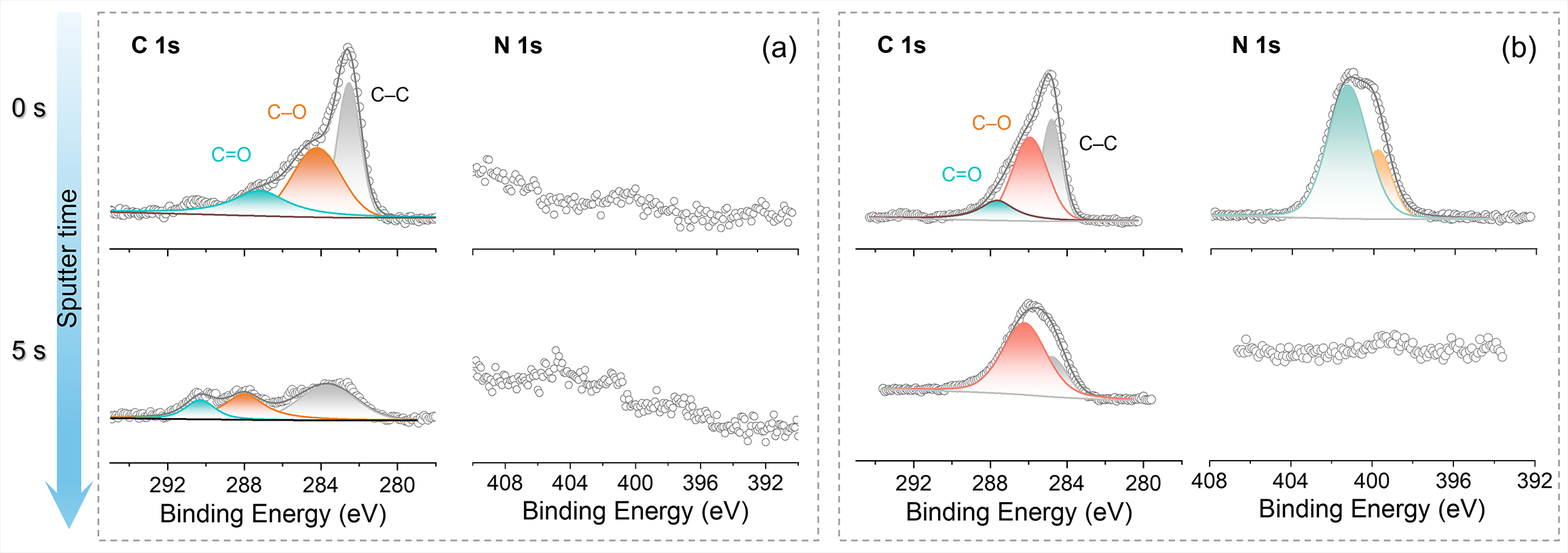


**Fig. S2** XPS depth profile of Zn anodes after 20 cycles at 1 mA cm^-2^ for 1 mAh cm^-2^ in (**a**) ZS and (**b**) Pah^+^


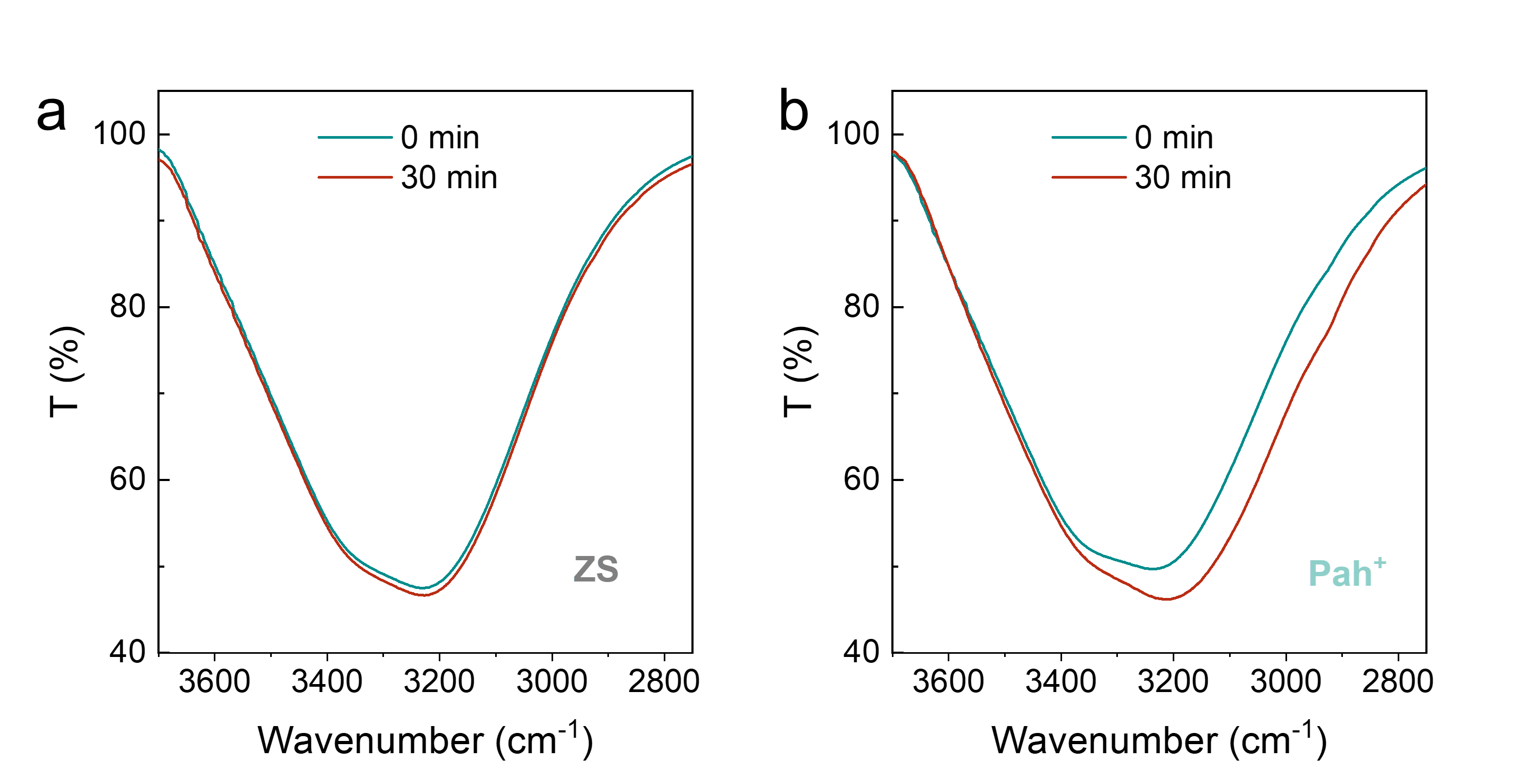


**Fig. 3** *In situ* FTIR spectroscopy of Zn anode surfaces during charging at 0.5 mA cm^−2^ in (**a**) ZS and (**b**) Pah^+^ electrolytes


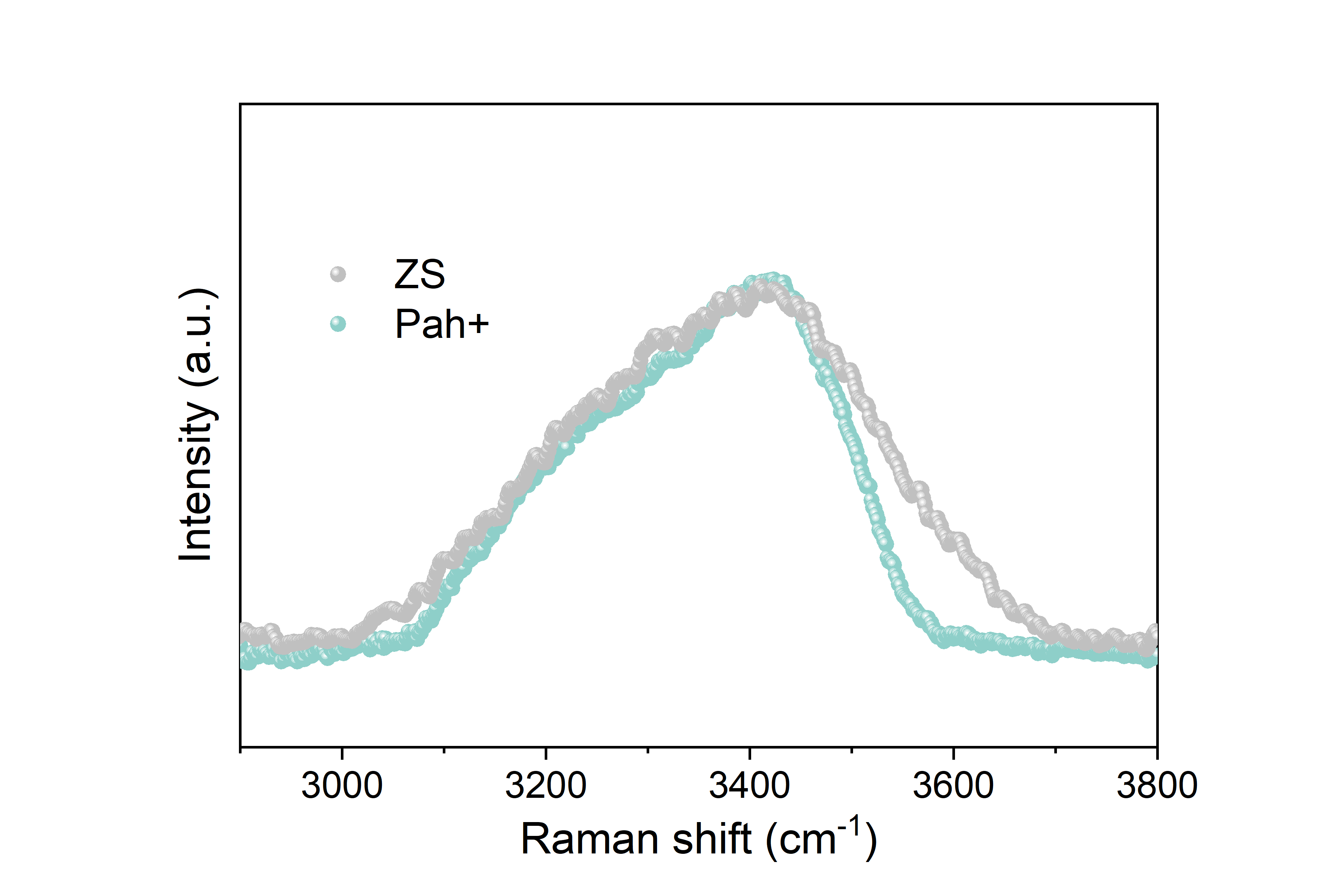


**Fig. S4** Raman spectra of Zn anode surfaces in different electrolytes


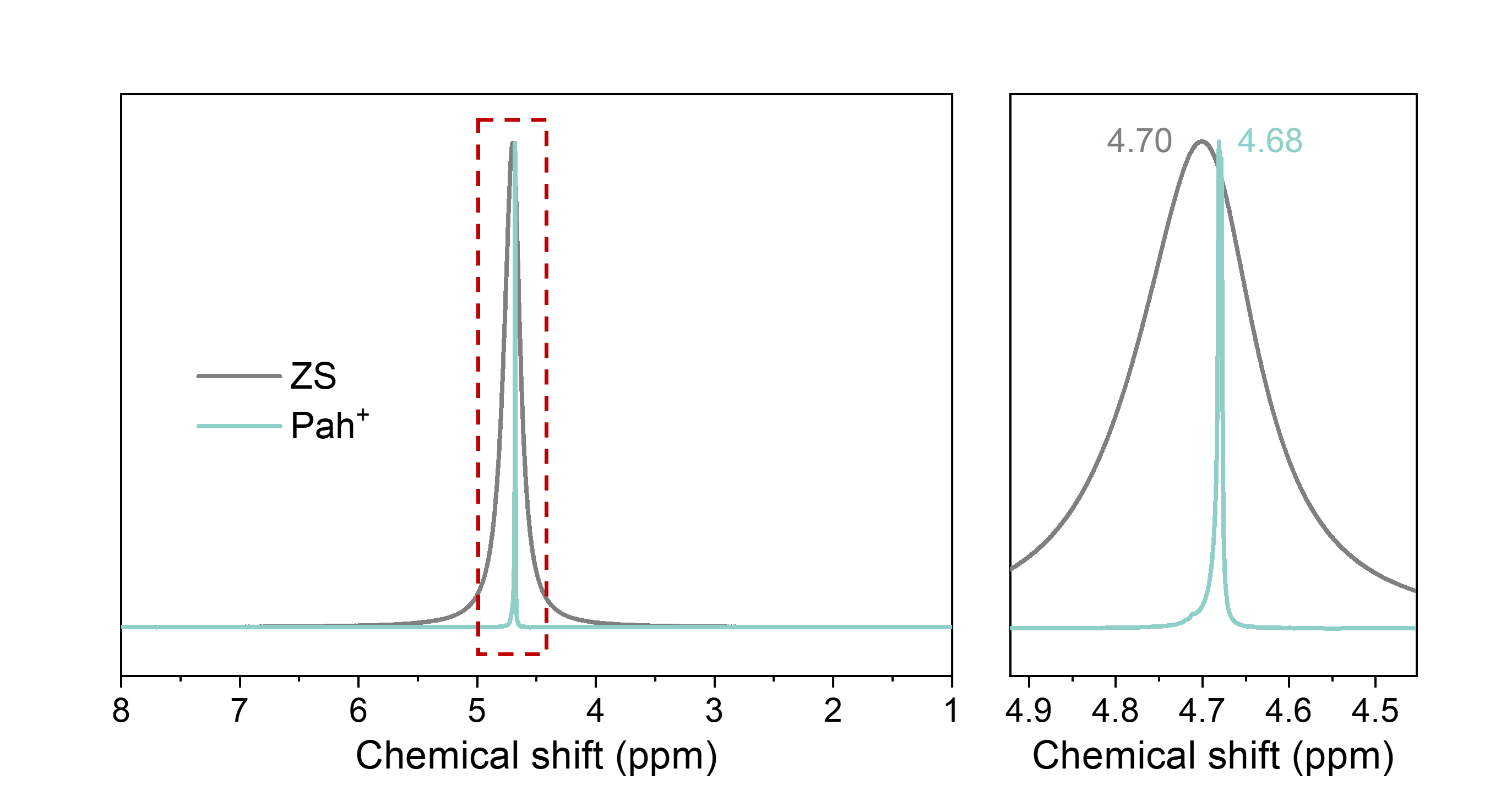


**Fig. S5** ^1^H NMR spectra of different electrolytes in D_2_O


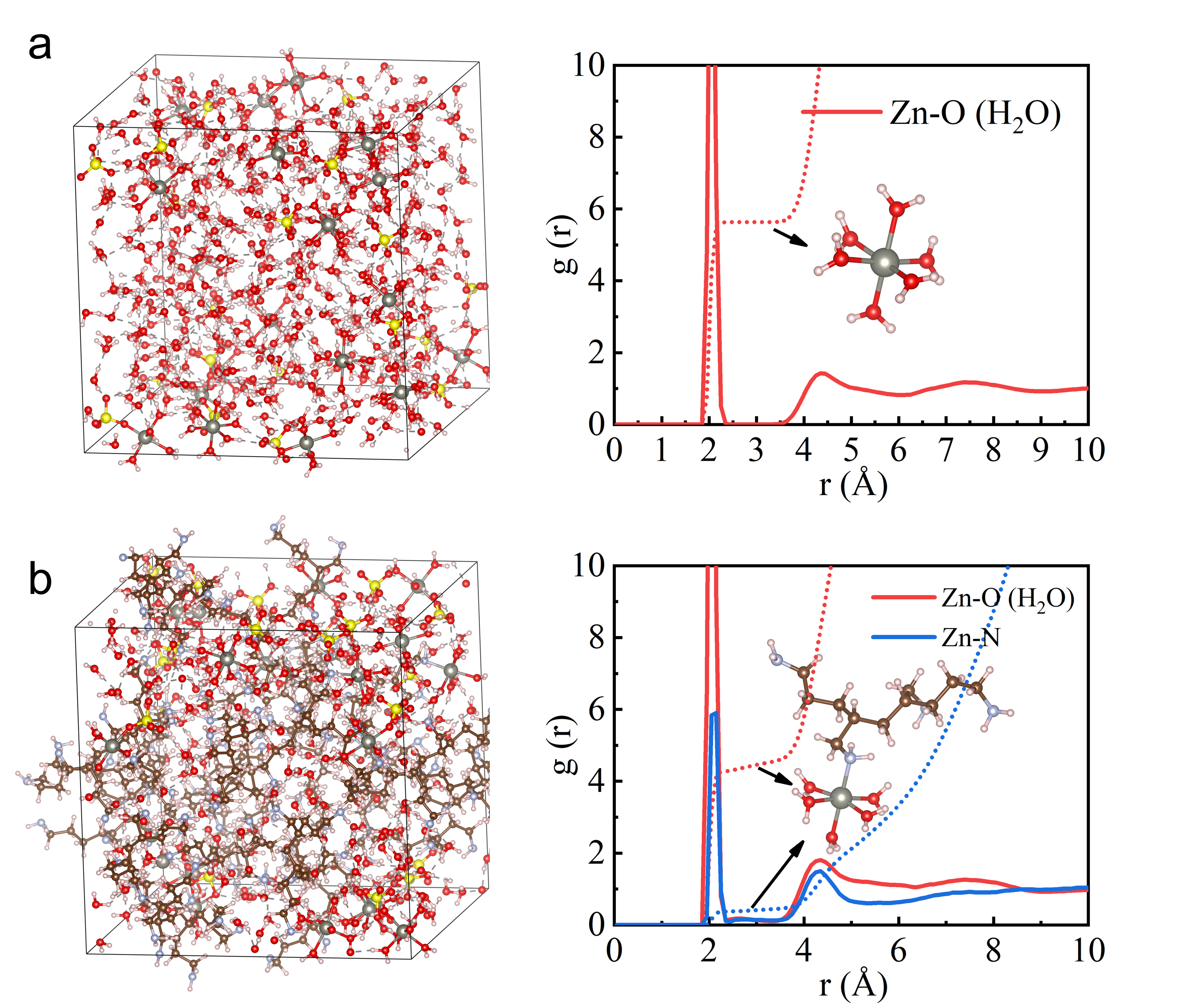


**Fig. S6** MD simulation and calculated RDF of (a) ZS and (b) Pah^+^ electrolytes


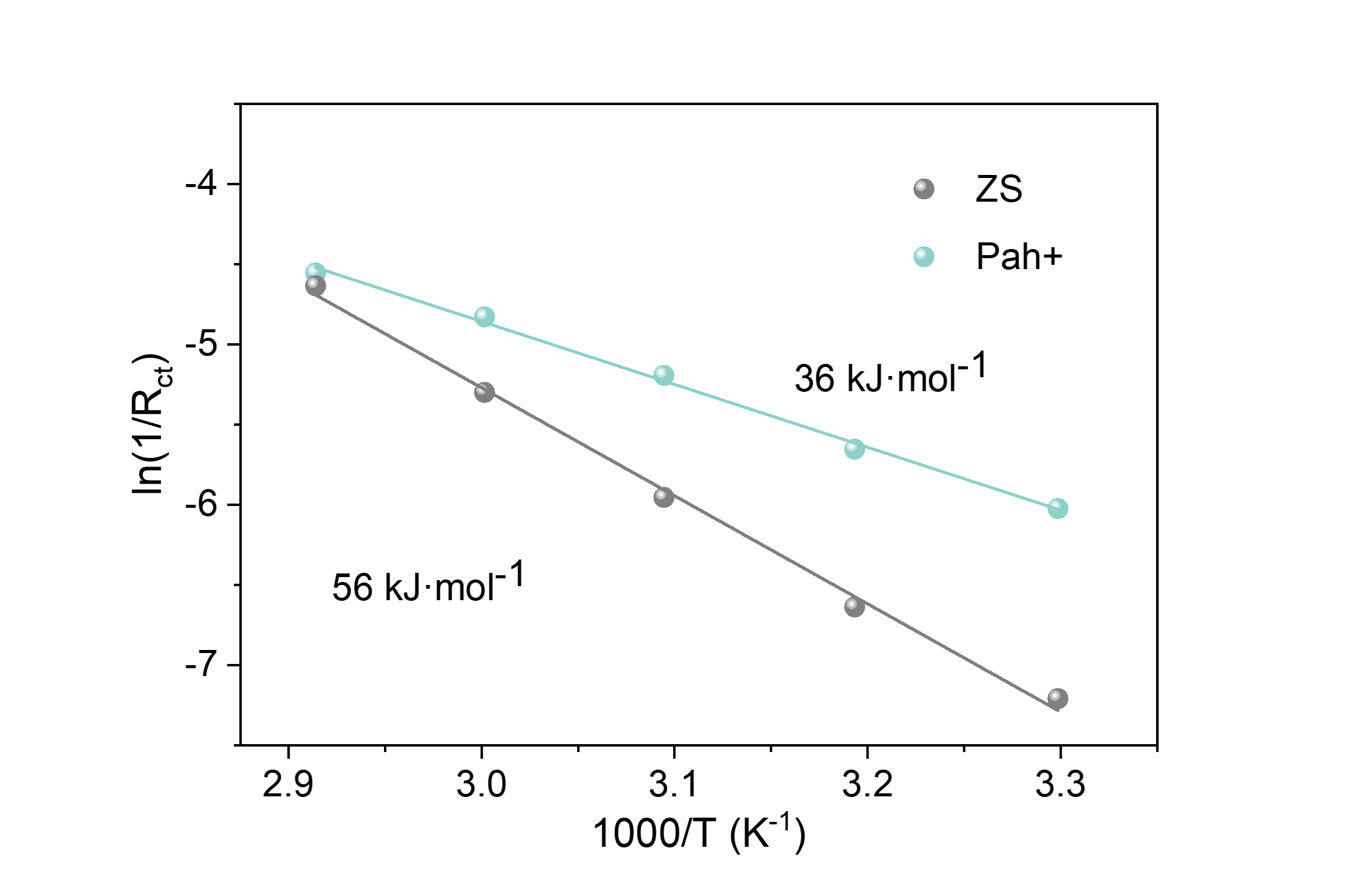


**Fig. S7** Fitted plots of R_ct_ measured at various temperatures based on the Arrhenius equation


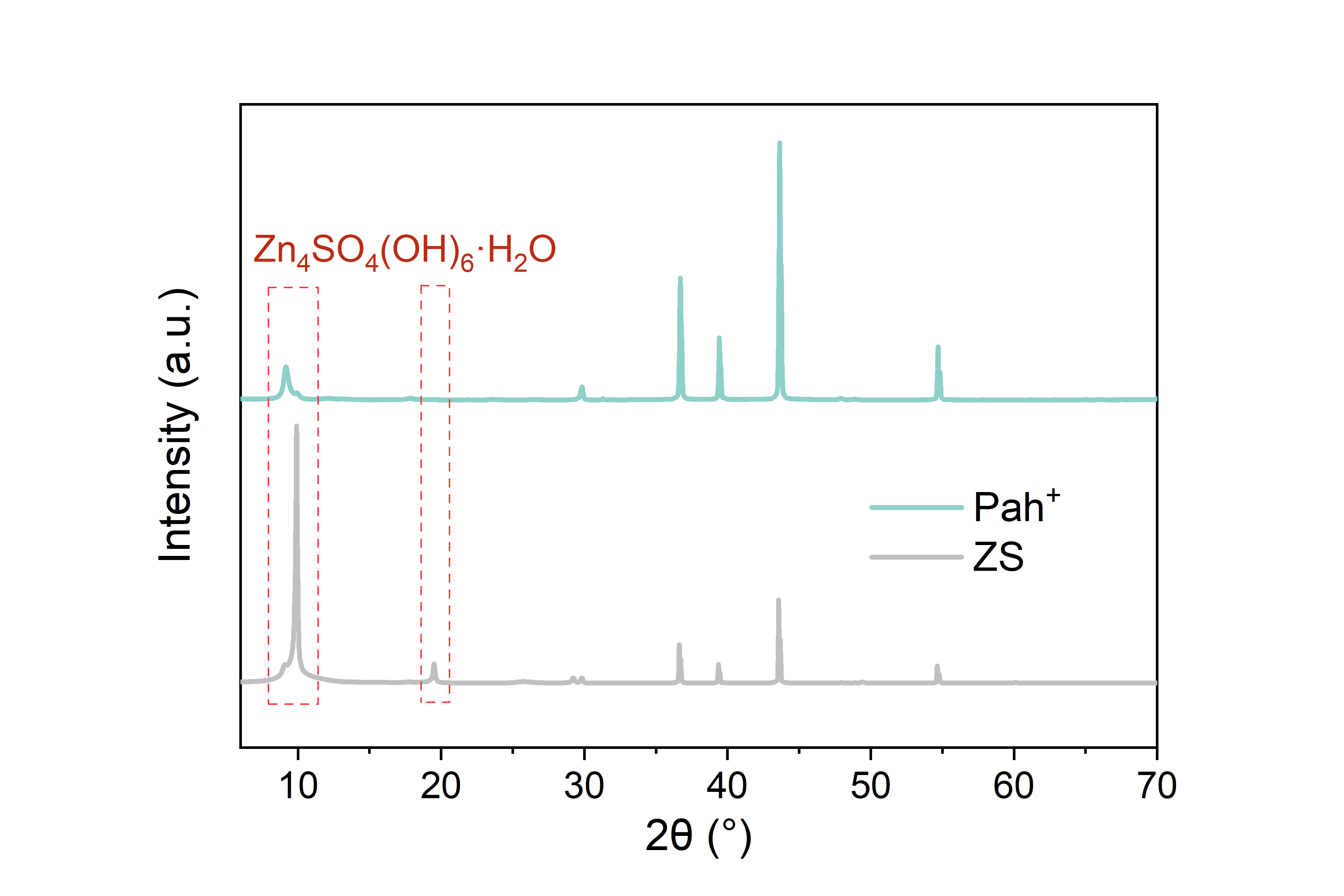


**Fig. S8** XRD patterns of Zn foils soaked in different electrolytes for 7 days


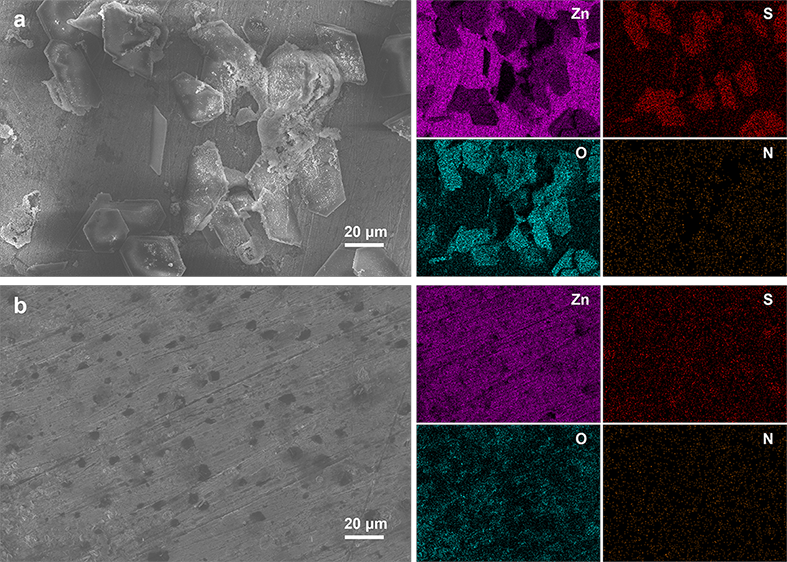


**Fig. S9** SEM and corresponding EDS mapping of Zn foils soaked in (**a**) ZS and (**b**) Pah^+^ for 7 days


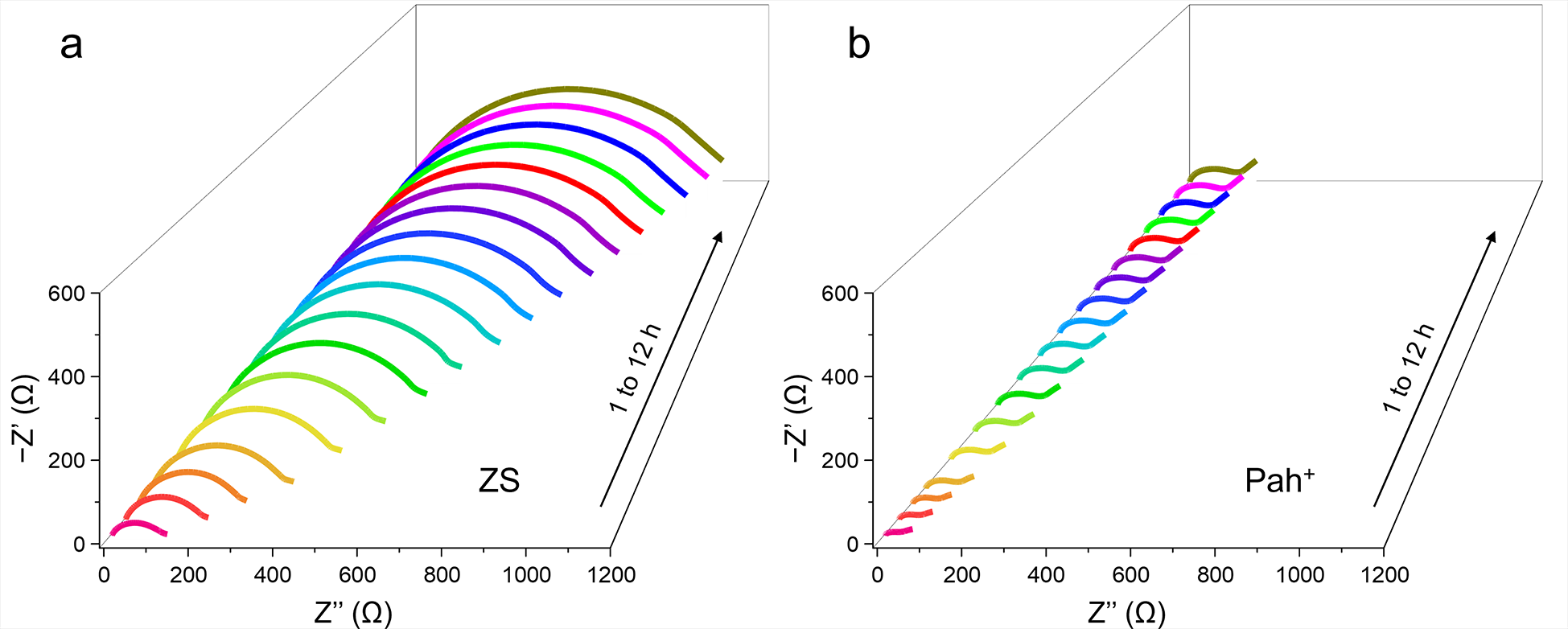


**Fig. S10** EIS Nyquist plots of Zn||Zn symmetric cells during the aging process from 1 to 12 h in (**a**) ZS and (**b**) Pah^+^


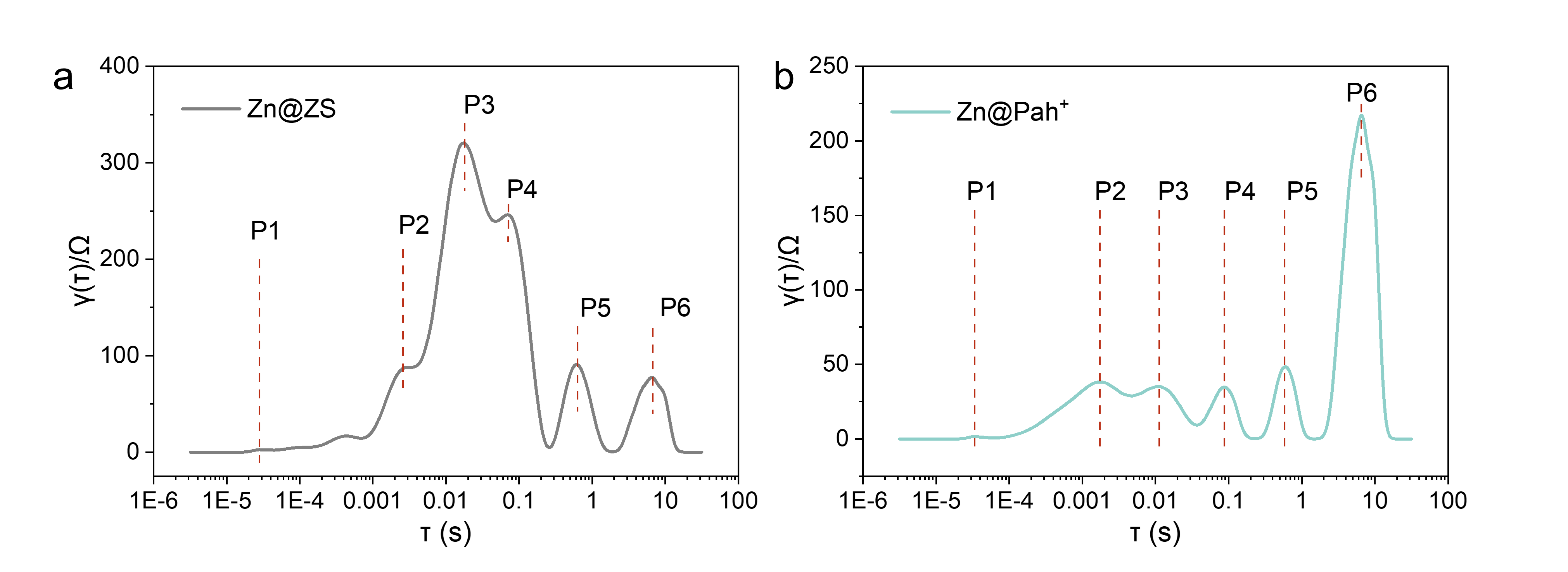


**Fig. S11** Deconvoluted DRT profiles for Zn||Zn symmetric cells aged for 12 h in two types of electrolytes

DRT plots of Zn||Zn symmetric cells revealed six characteristic peaks with different time constants [S1]:

P1: at ~ 0.035 ms, relaxation of electrons.

P2: at ~ 0.002 s, adsorption of Zn(H_2_O)_6_^2−^.

P3: at ~0.011 s, desolvation of Zn(H_2_O)_6_^2−^.

P4: at ~0.08 s, migration and crystallization of Zn ions and atoms on the electrode.

P5: at ~0.6 s, charge transfer across the interfaces.

P6: at ~6.5 s, mass transfer.


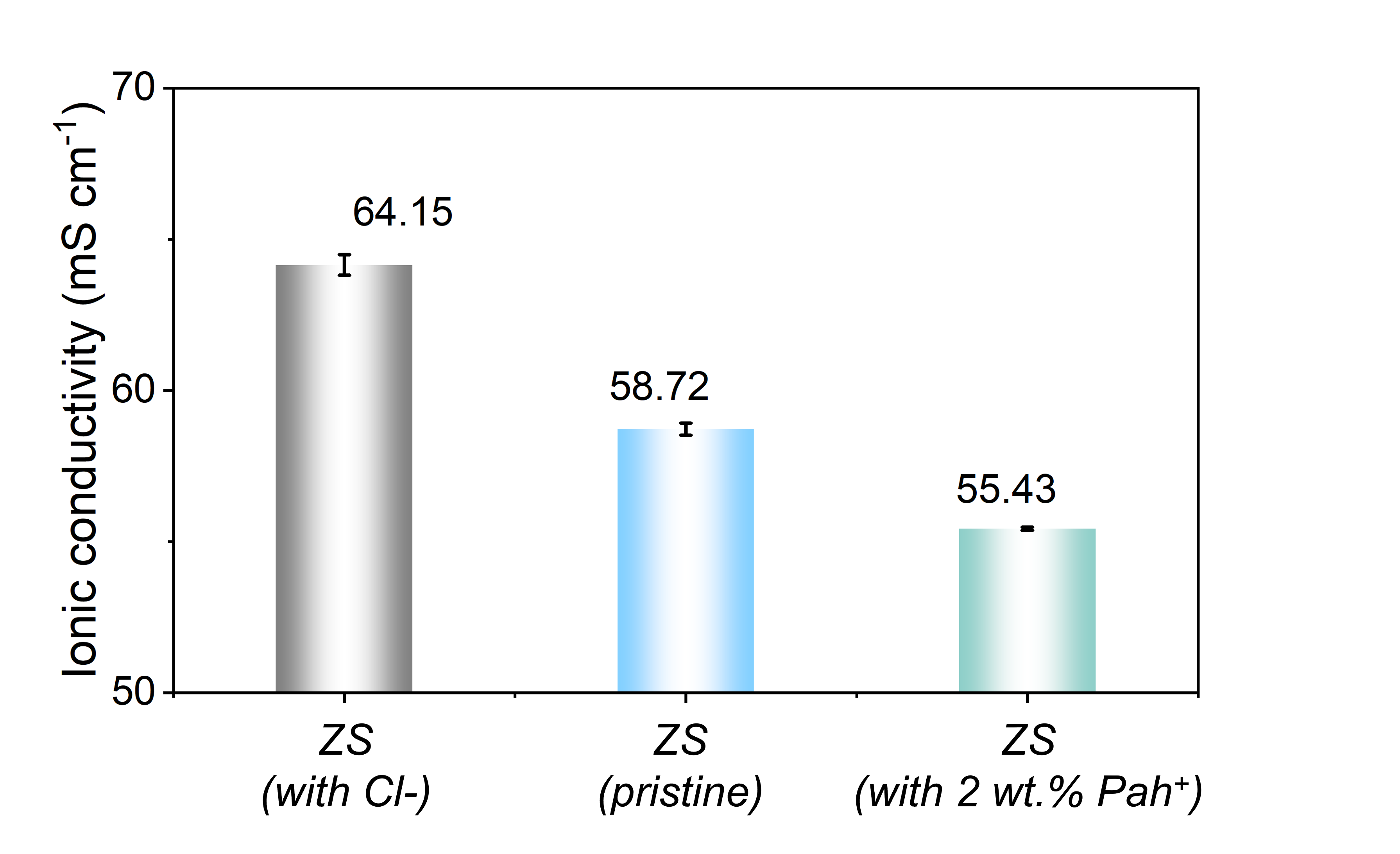


**Fig. S12** Ionic conductivity of different electrolytes

ZS (with Cl^−^) refers to the 2 M ZnSO_4_ containing ZnCl_2_ additive, which is the controlled sample studied in this work.

ZS (pristine) refers to the pristine 2 M ZnSO_4_ and ZS (with 2 wt.% Pah^+^) refers to the 2 M ZnSO_4_ containing Pah^+^ additive.


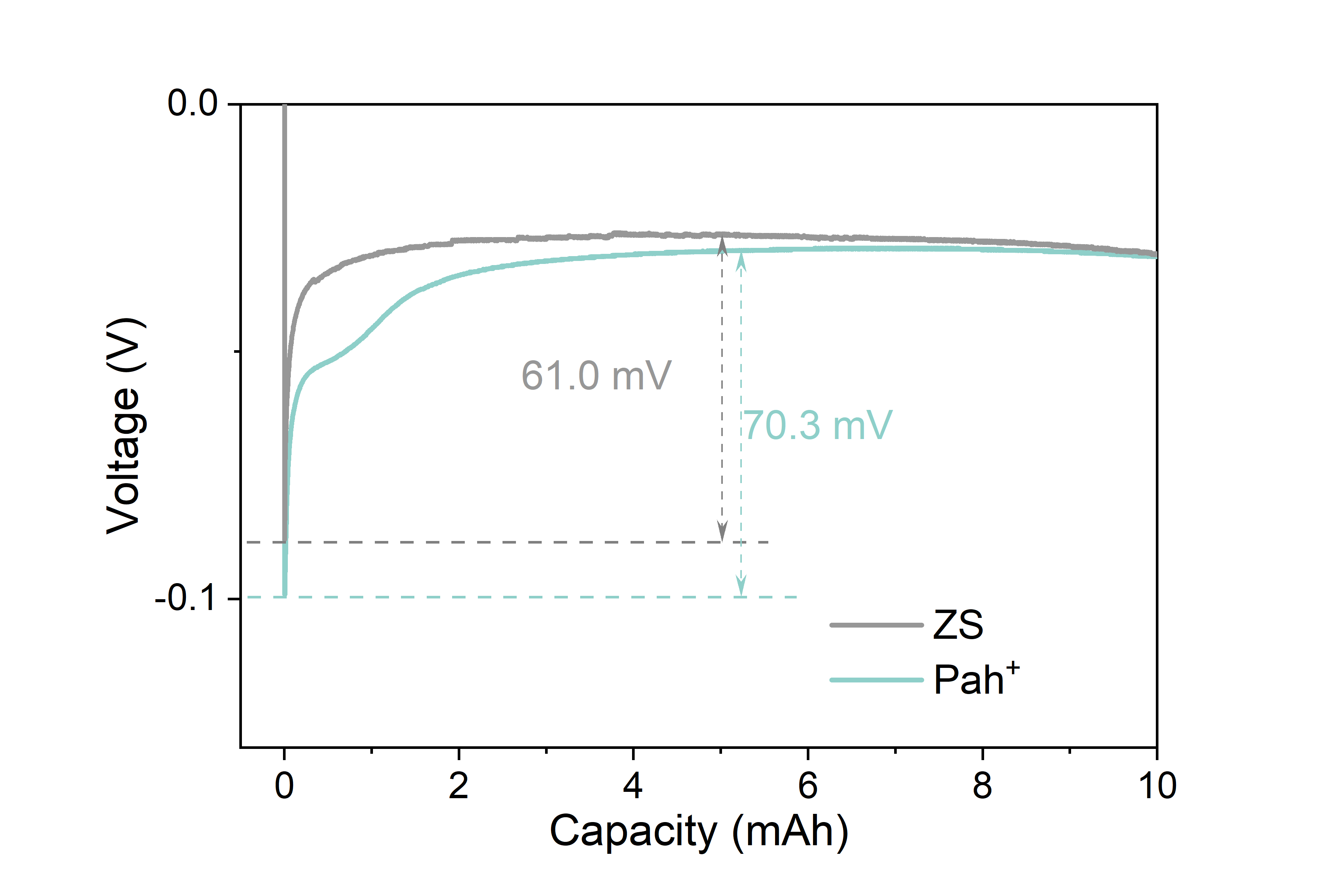


**Fig. S13** Galvanostatic discharge curves of Zn||Cu asymmetric cells in different electrolytes at 2 mA cm^−2^ for 10 mAh cm^−2^


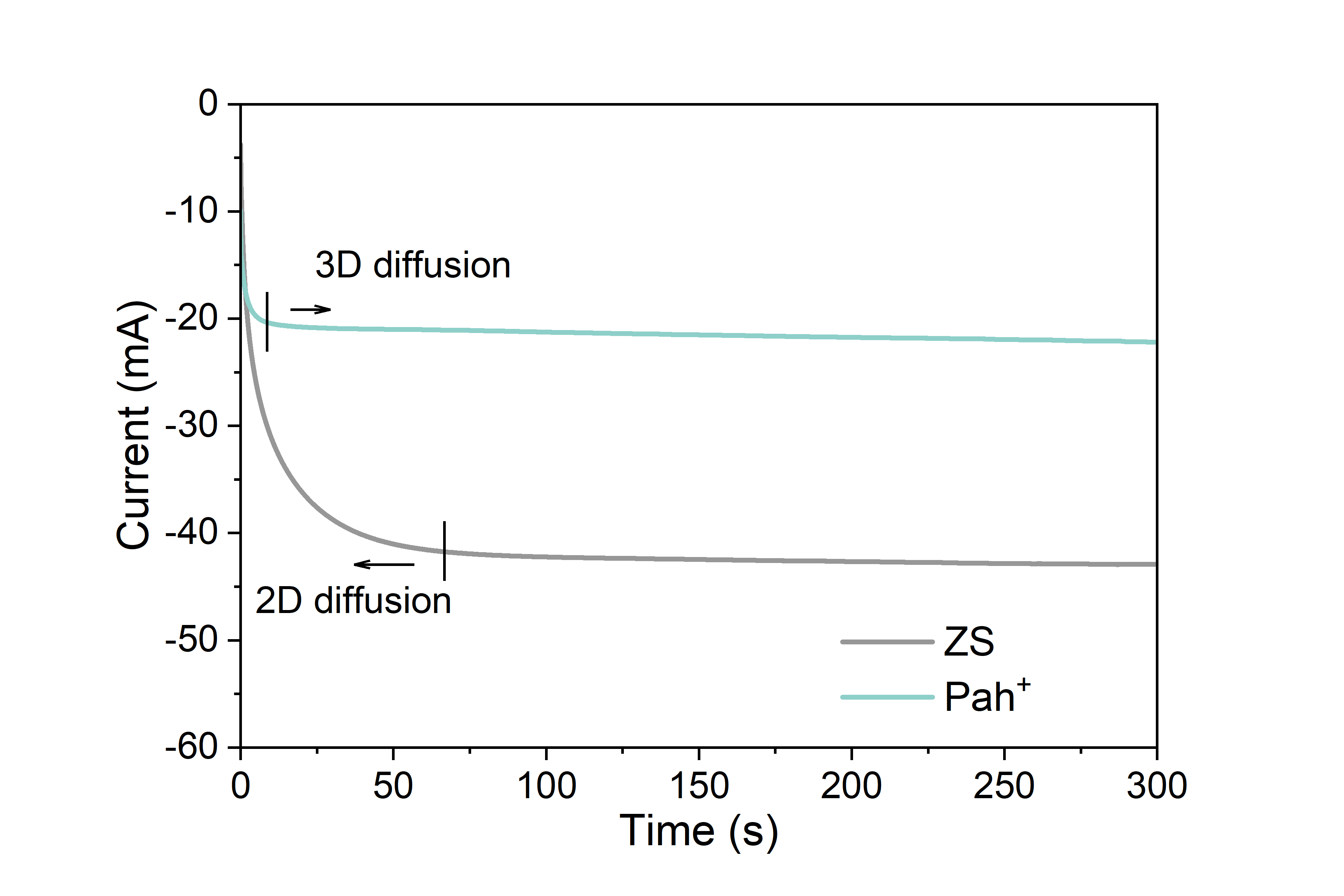


**Fig. S14** Chronoamperometry *i-t* curves of Zn||Zn cells in different electrolytes with −150 mV potential bias


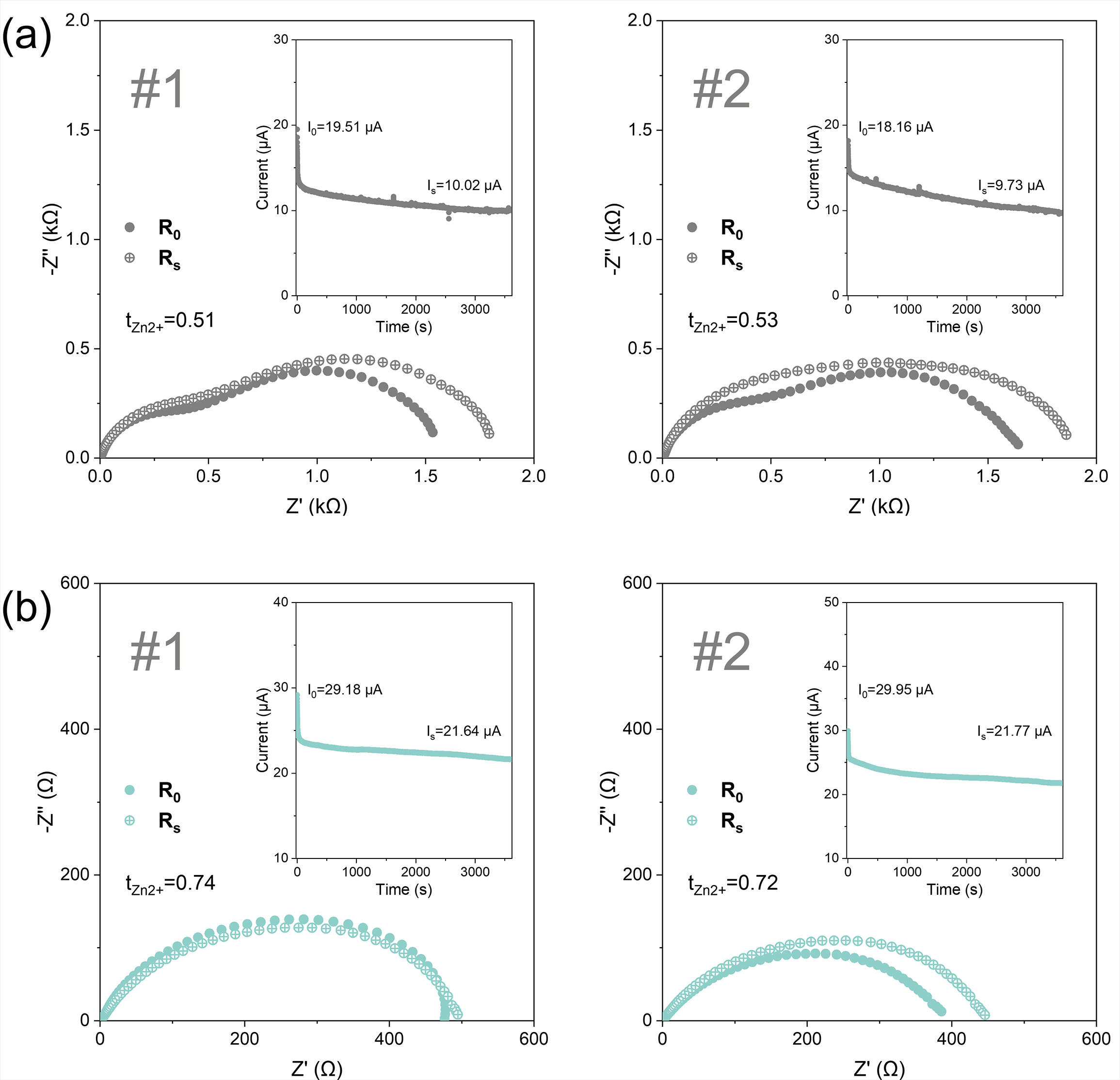


**Fig. S15** Transference number measurement for symmetric cells in (**a**) ZS and (**b**) Pah^+^ using the Bruce-Vincent method

The transference number of Zn^2+^ (*t*_Zn2+_) was calculated using the Bruce-Vincent method by fitting with the following equation:

$$t_{Zn2+}=\frac{I_{s}\left( \Delta V-I_{0}R_{0} \right)}{I_{0}\left( \Delta V-I_{s}R_{s} \right)}$$

Where the $I_{0}$ and $I_{s}$ refer to the current of the initial and steady states, $R_{0}$ and $R_{s}$ refer to the resistance of the initial and steady states, $\Delta V$ is the applied polarization voltage (20 mV).


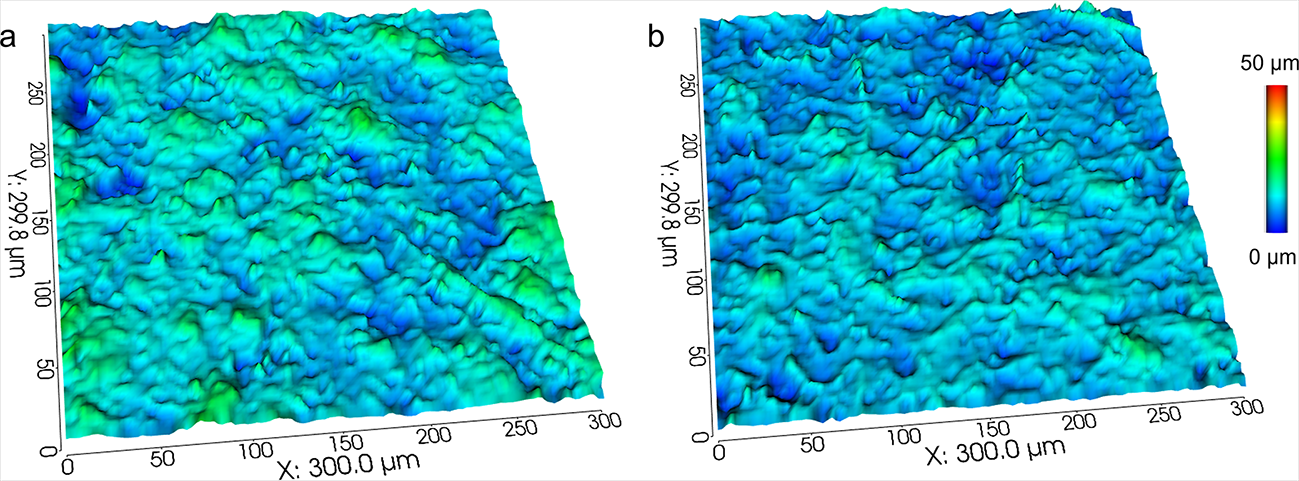


**Fig. S16** CLSM image of Zn deposits in Zn||Zn symmetric coin cells at 2 mA cm^−2^ for 10 mA cm^−2^ in (**a**) ZS and (**b**) Pah^+^


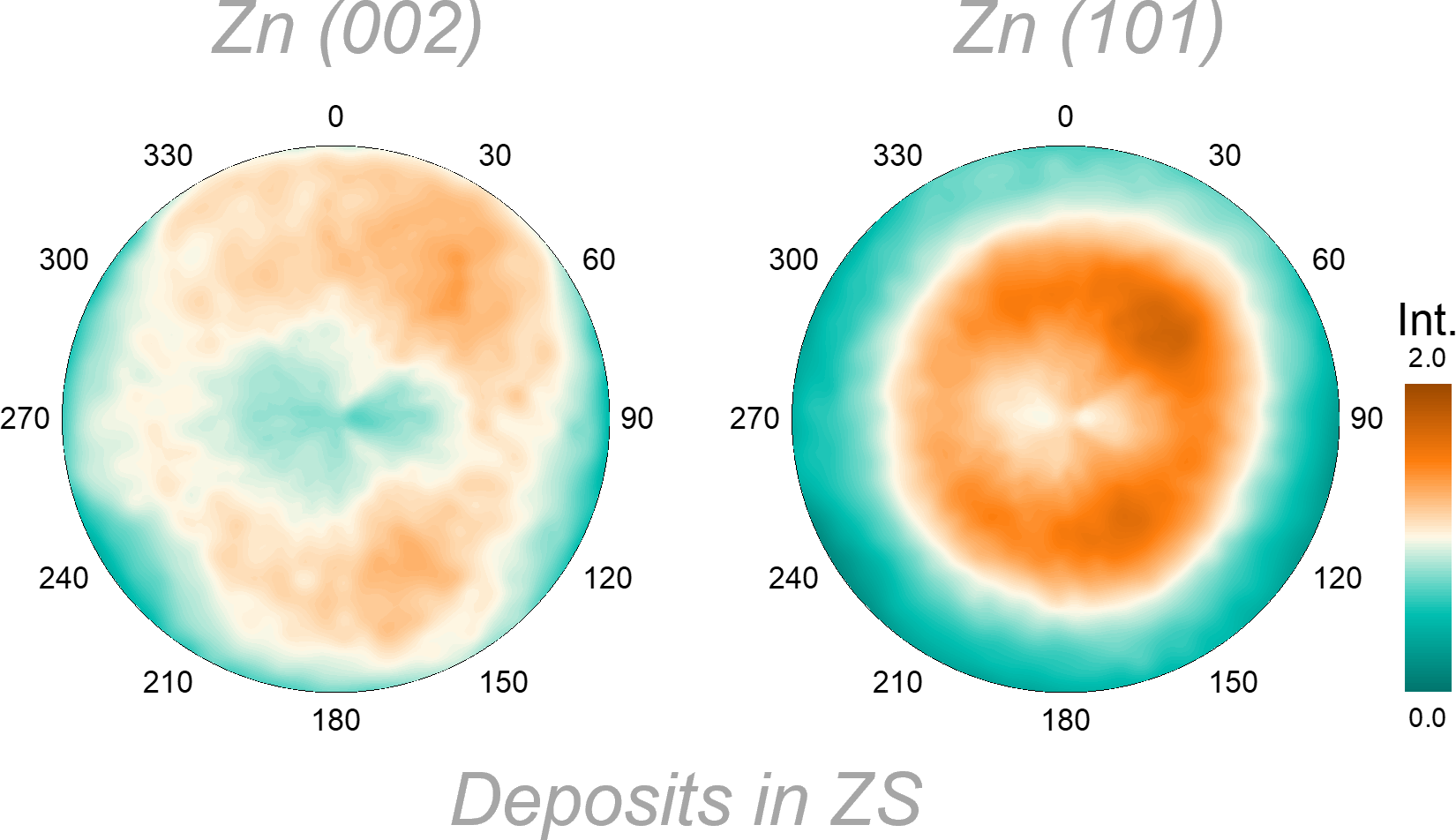


**Fig. S17** XRD pole figures of Zn deposits in Zn||Zn symmetric coin cells with ZS electrolyte


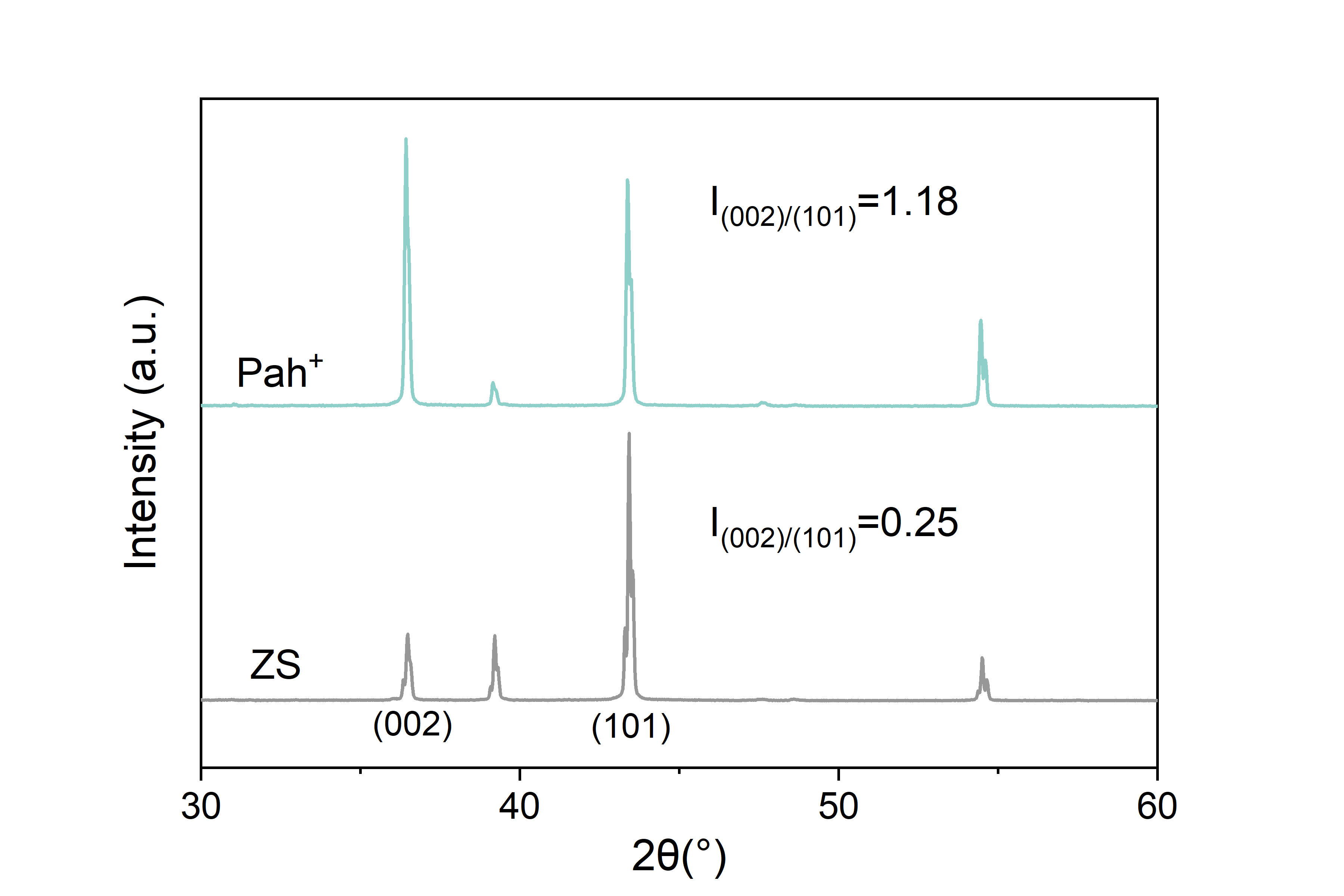


**Fig. S18** XRD patterns of Zn deposits in different electrolytes


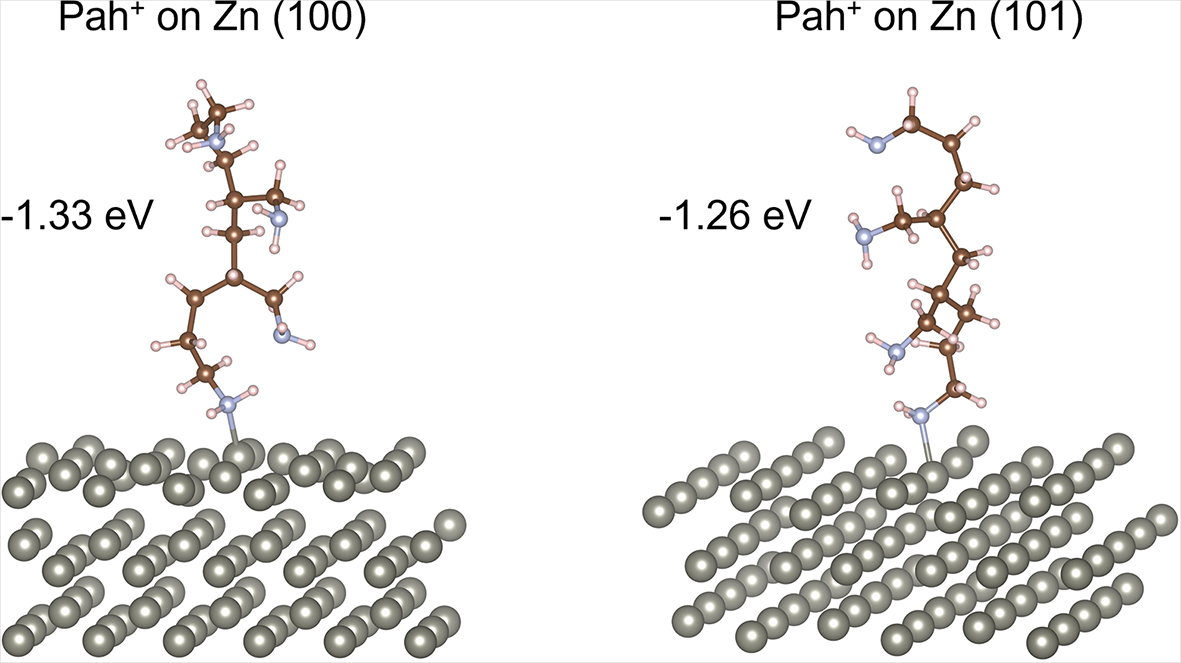


**Fig. S19** Adsorption of Pah^+^ on Zn (100) and (101) surfaces


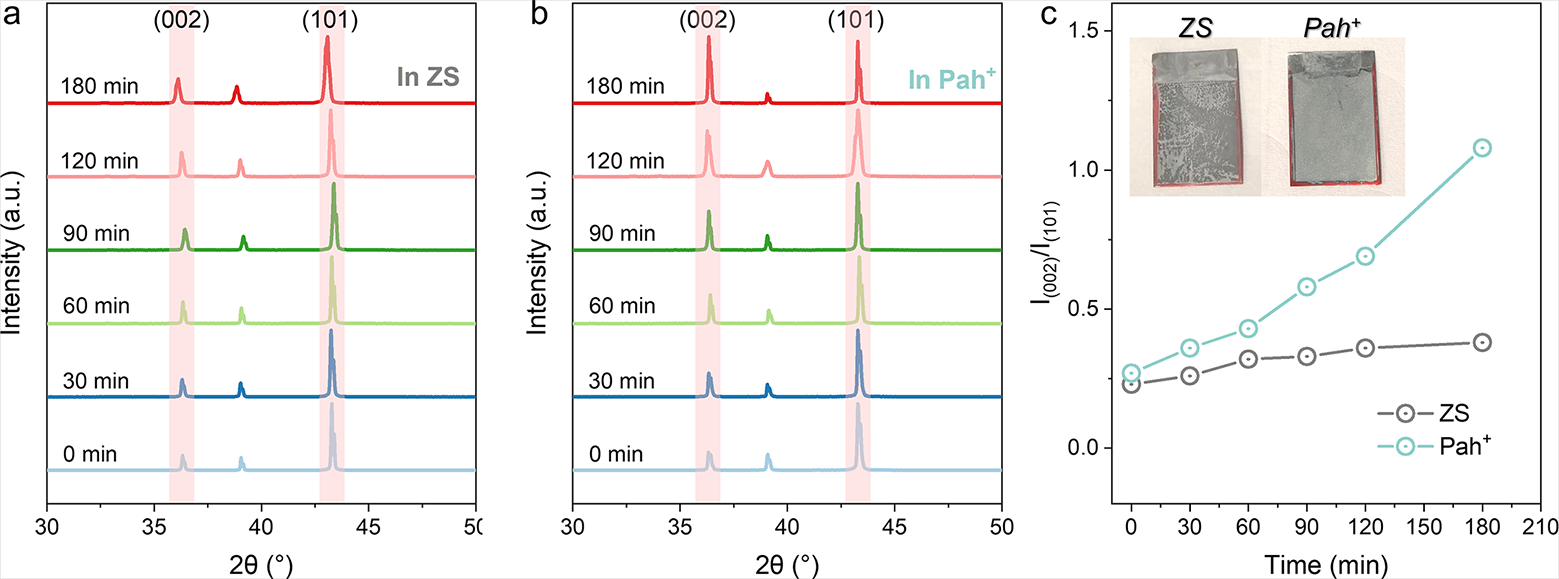


**Fig. S20** XRD patterns of Zn anodes during different deposition time with the current of 5 mA cm^−2^ in (**a**) ZS and (**b**) Pah^+^, and (**c**) the intensity ratio between Zn (002) and (101) facets (inserted with the digital photos of Zn anodes after deposition)


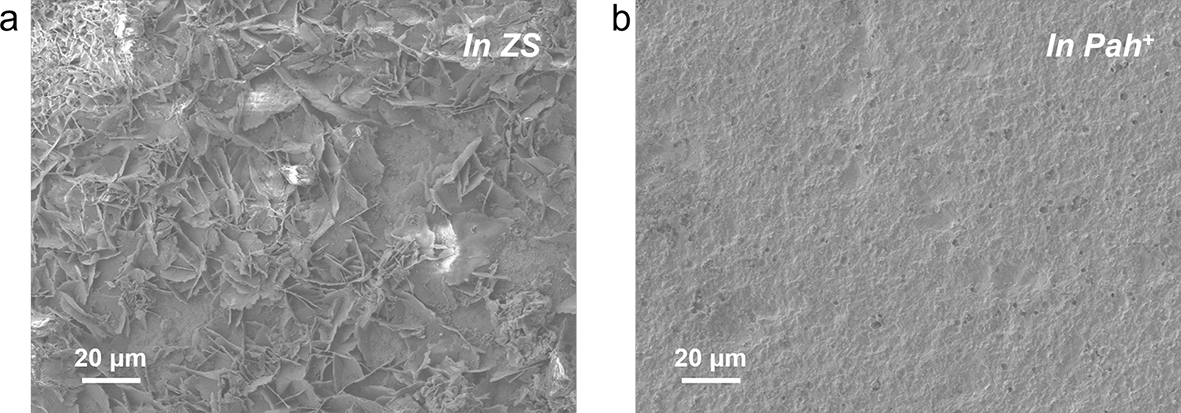


**Fig. S21** SEM images of Zn anodes in (**a**) ZS and (**b**) Pah^+^ after 20 cycles at 1 mA cm^−2^ for 1 mAh cm^−2^


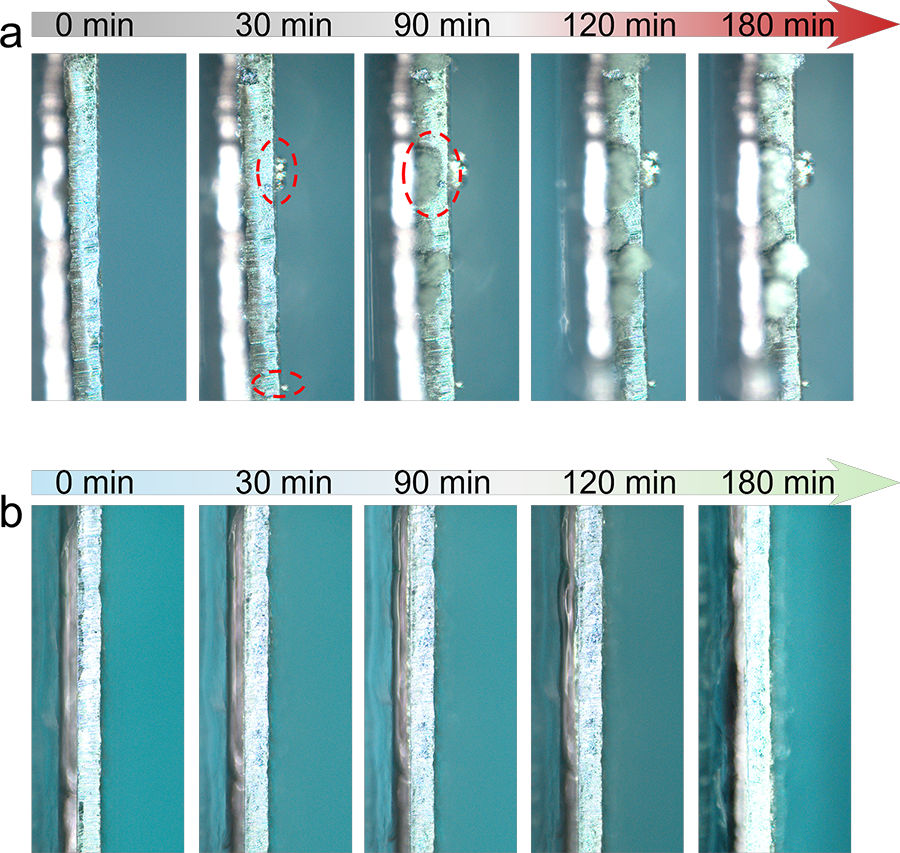


**Fig. S22** *In situ* optical microscopy images of Zn plates deposited in (a) ZS and (b) Pah^+^ at fixed current of 2 mA cm^−2^ for a duration of 180 minutes


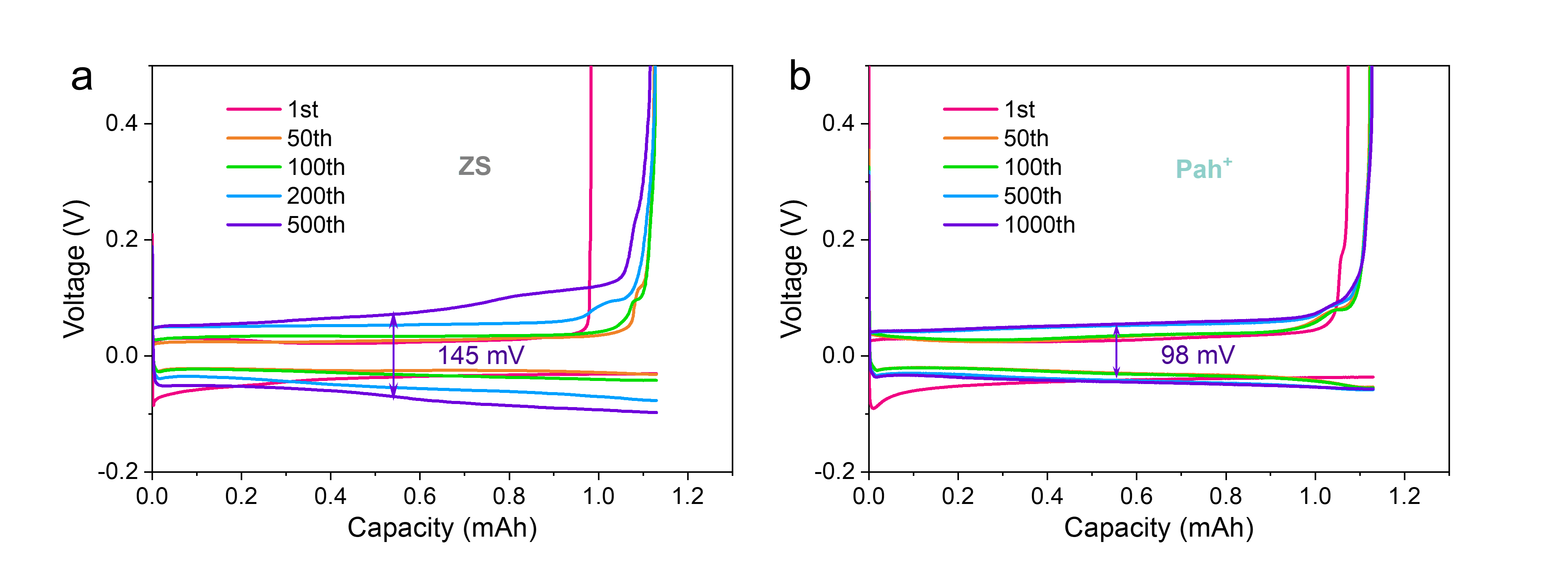


**Fig. S23** Voltage profiles of Zn||Cu asymmetric cell in (**a**) ZS and (**b**) Pah^+^


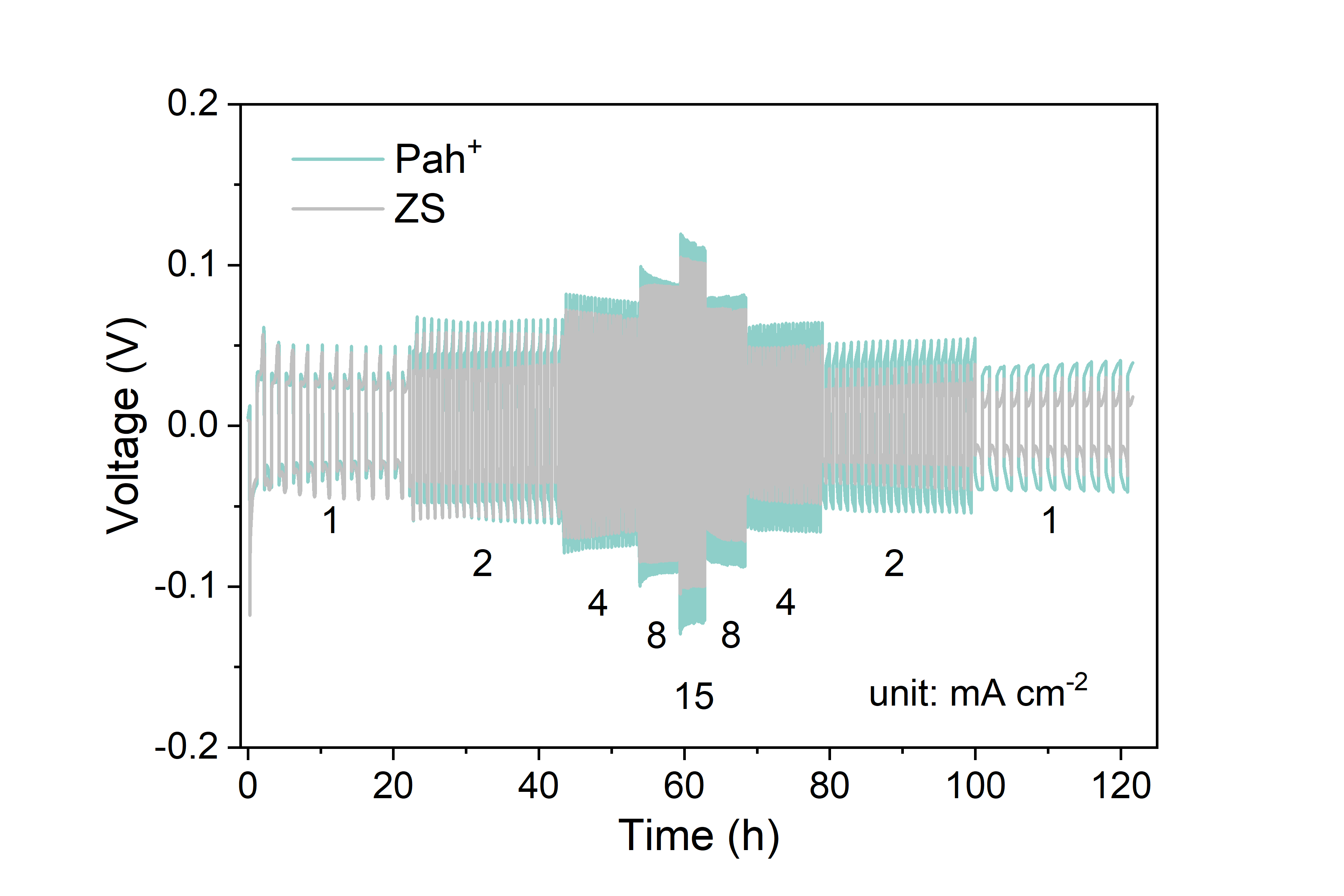


**Fig. S24** Rate capability of Zn||Zn cells


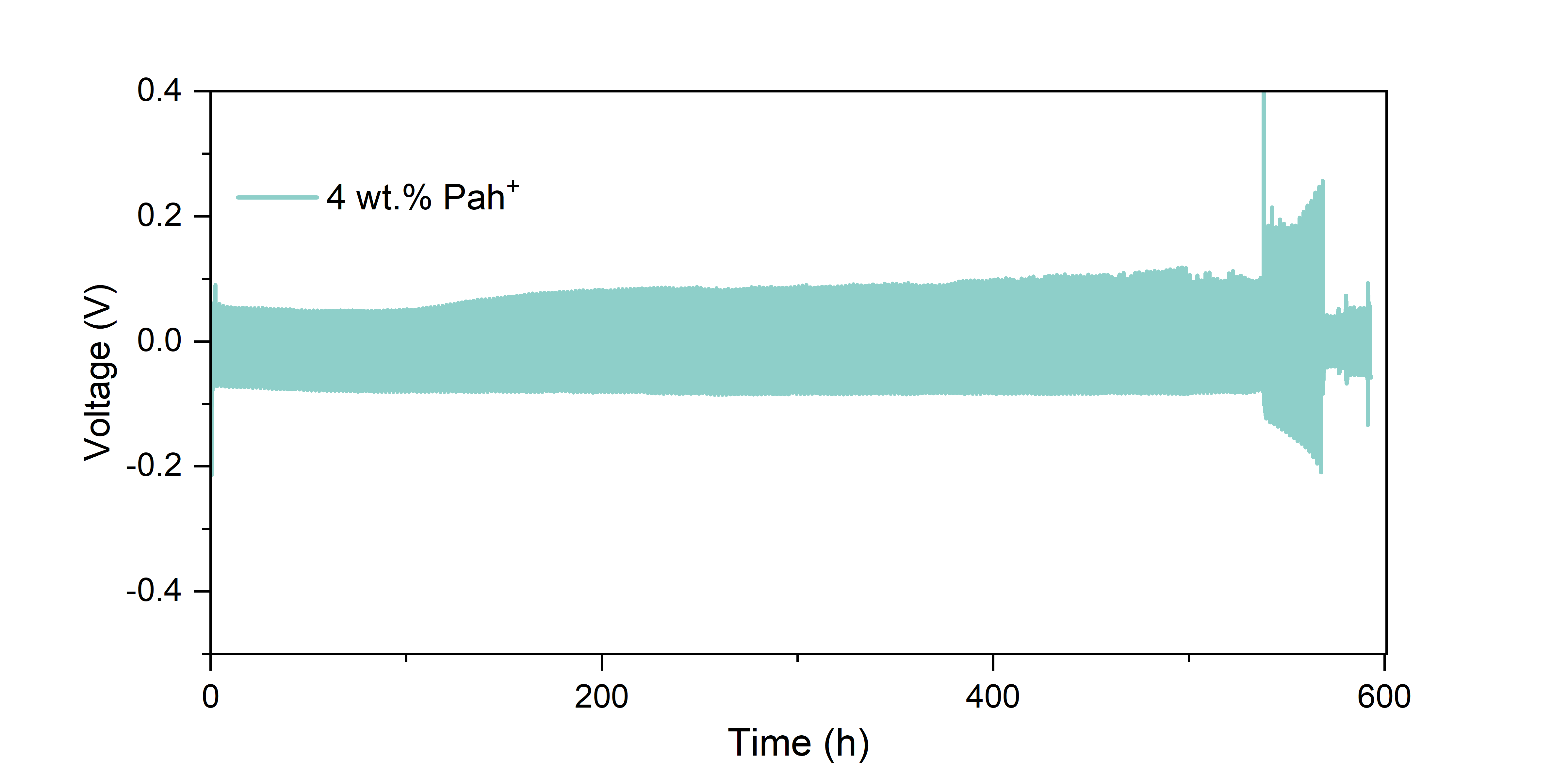


**Fig. S25** Cycling performance of Zn||Zn symmetric cell in 4 wt.% Pah^+^ at 5 mA cm^−2^ for 5 mA cm^−2^


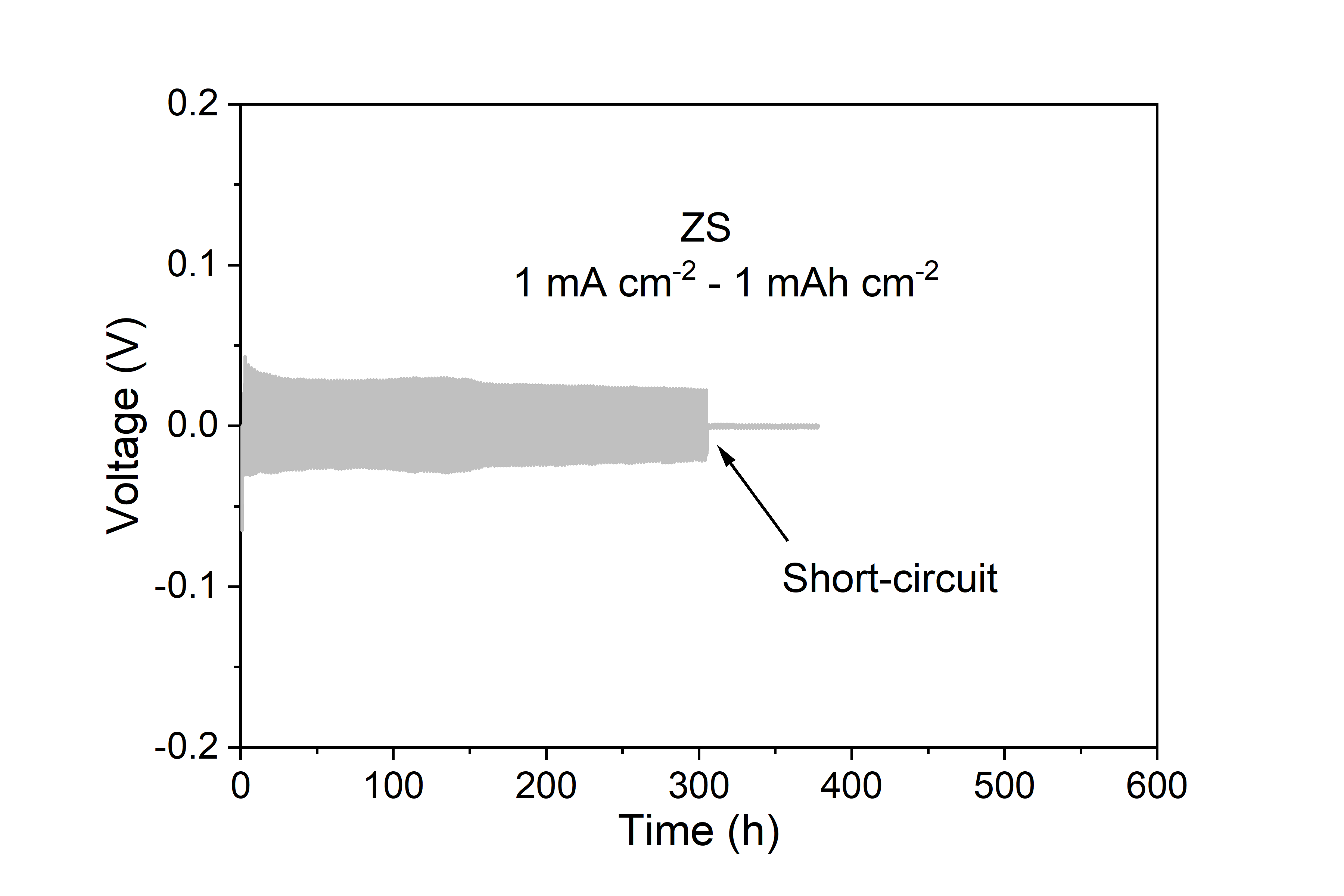


**Fig. S26** Cycling performance of Zn||Zn symmetric cell in ZS at 1 mA cm^−2^ for 1 mA cm^−2^


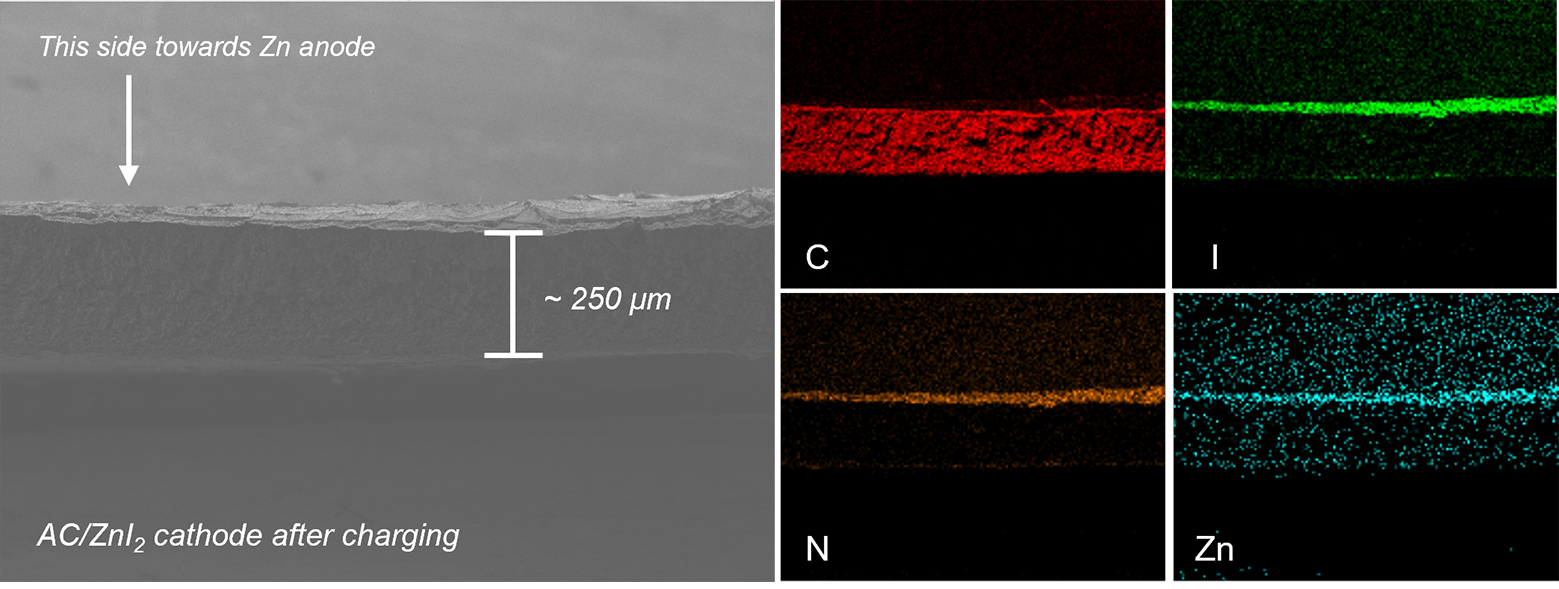


**Fig. S27** SEM image and the EDS mapping of the cathode cross section

The observed N signals are attributed to the adsorption of Pah^+^ on the cathode surface, where it interacts with iodine species to immobilize them and suppress their shuttling.


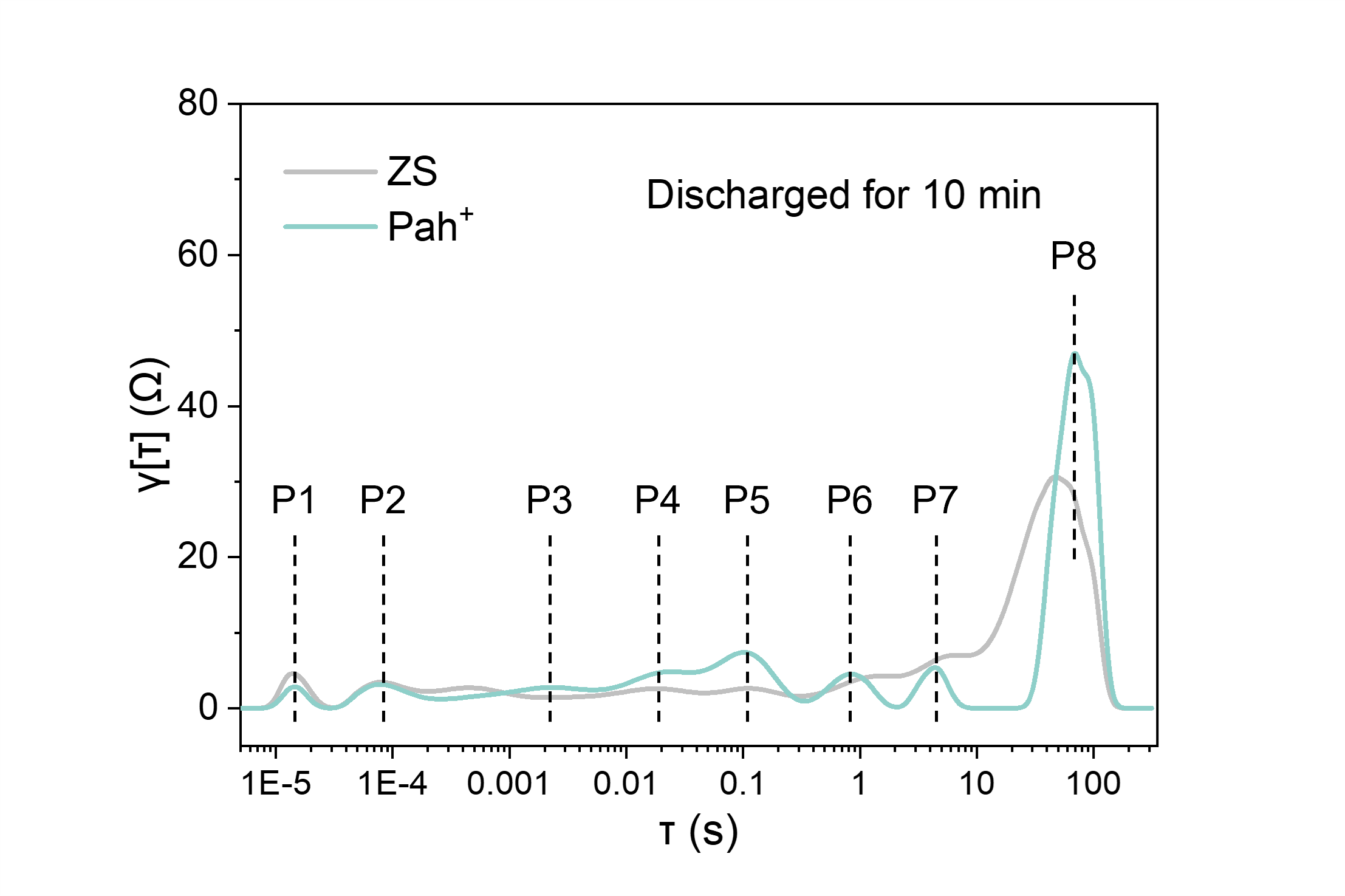


**Fig. S28** DRT plots of Zn-iodine batteries after discharging at 1 mA cm^−2^ for 10 min

P1: relaxation of electrons at Zn metal anode.

P2: relaxation of electrons at iodine cathode.

P3: adsorption of Zn(H_2_O)_6_^2−^.

P4: desolvation of Zn(H_2_O)_6_^2−^.

P5: migration and crystallization of Zn ions and atoms.

P6: charge transfer across the interfaces.

P7: mass transfer process.

P8: diffusion of polyiodide species.


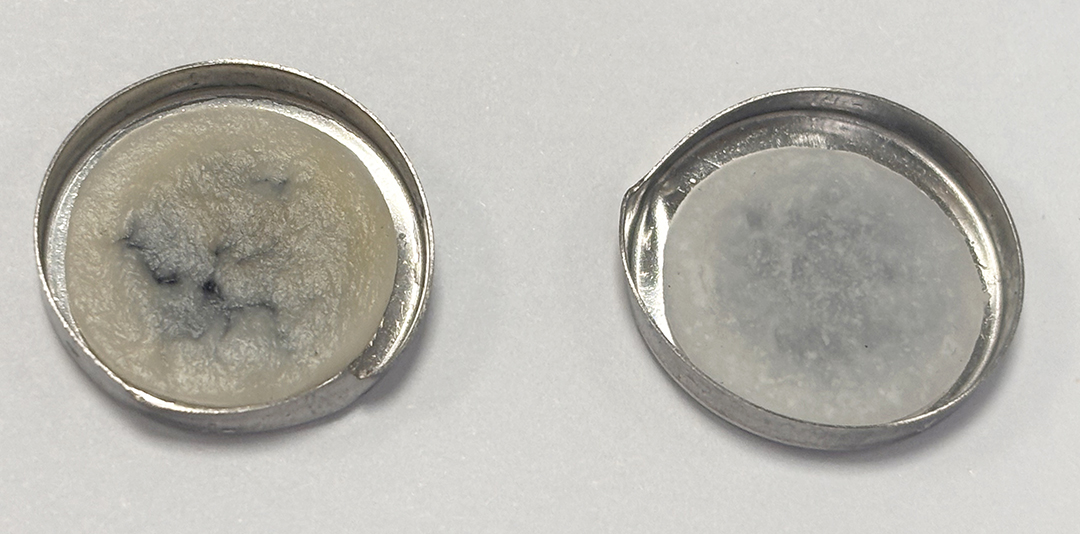


**Fig. S29** Optical images of the membranes in (left) ZS and (right) Pah^+^ after the discharging process at 1 mA cm^−2^ for 60 minutes

(Observed from the Zn metal anode side, below the membrane is the iodine cathode.)


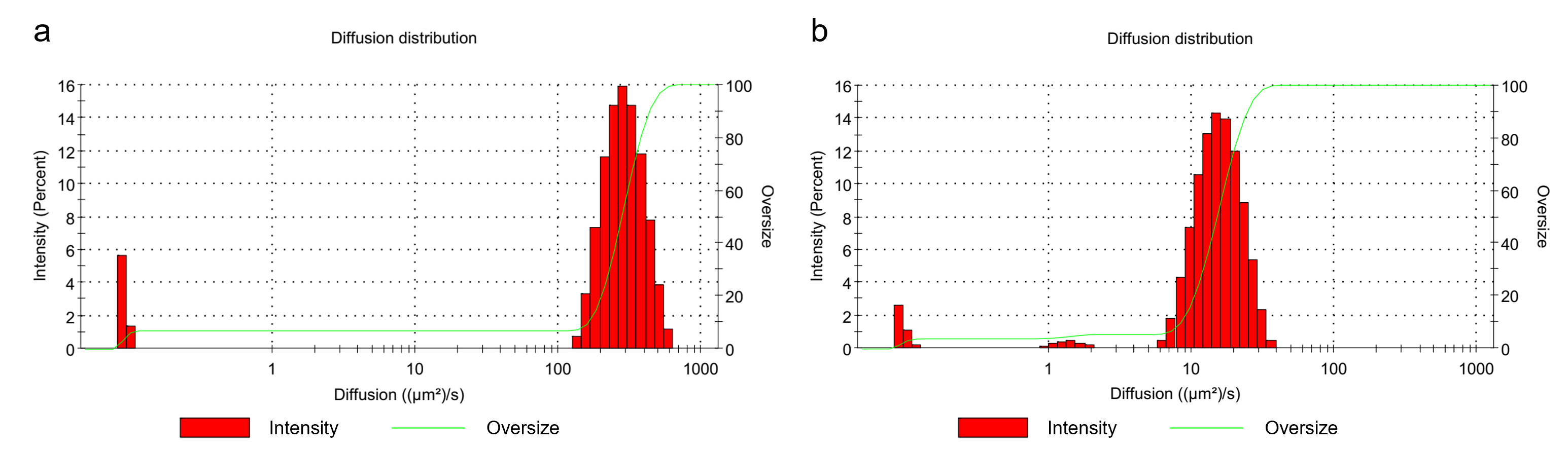


**Fig. S30** Calculated diffusion distribution of (**a**) ZS and (**b**) Pah^+^ electrolyte by DLS measurement


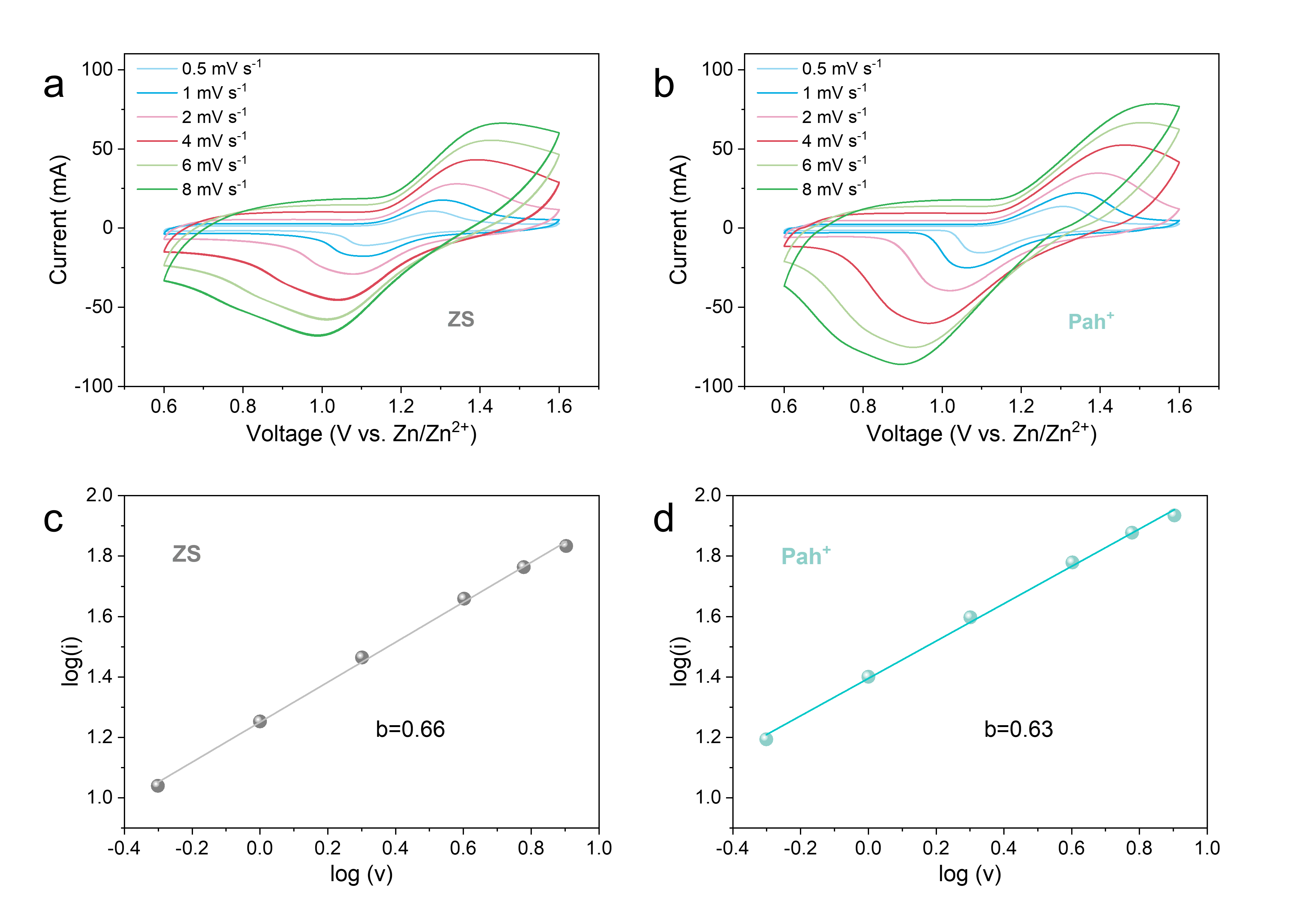


**Fig. S31** CV profiles of Zn-iodine batteries within the voltage of 0.6 to 1.6 V at different scan rates in (**a**) ZS and (**b**) Pah^+^, and the linear fitting of log (v) versus log (i) of batteries with (**c**) ZS and (**d**) Pah^+^


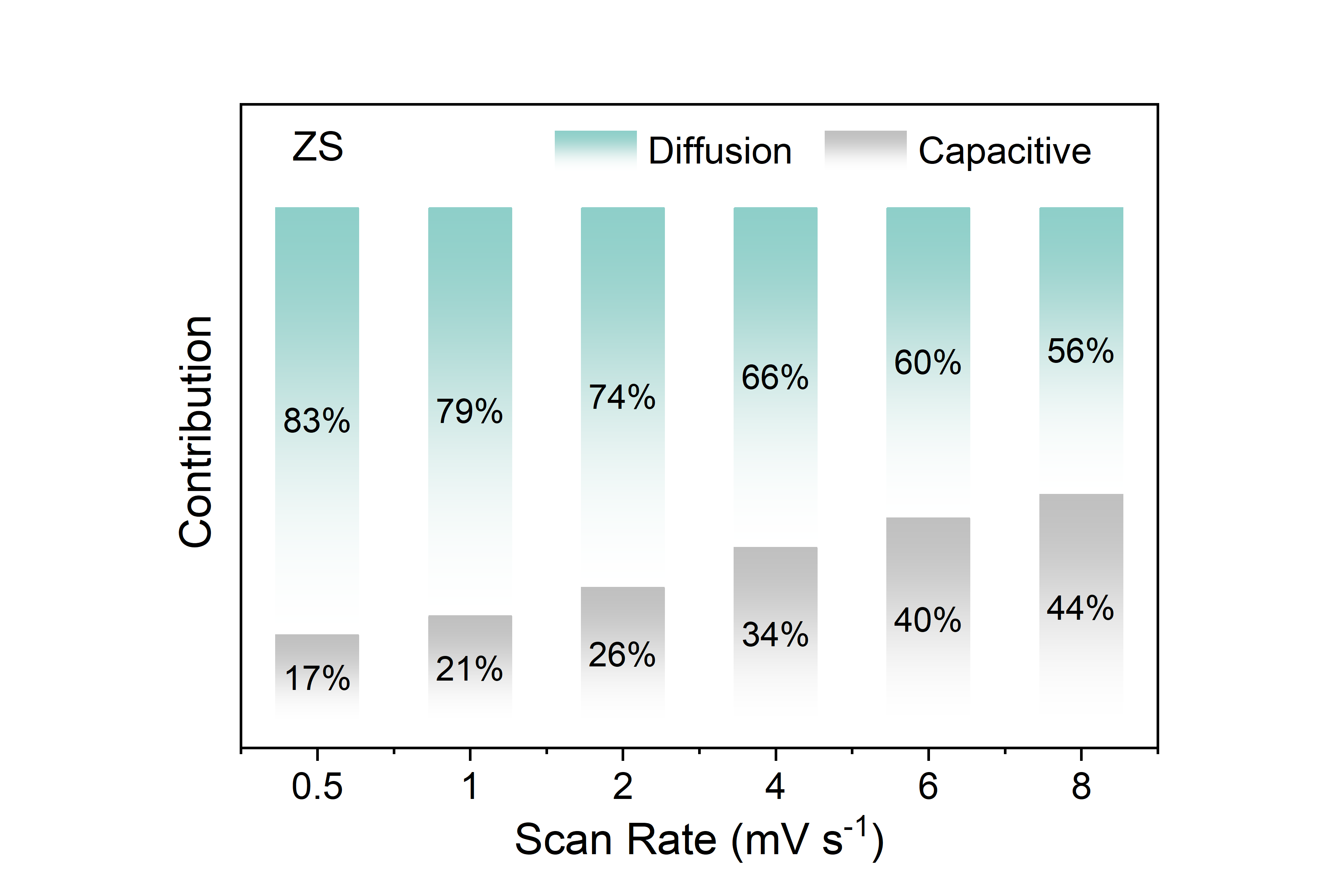


**Fig. S32** Calculated capacity contribution at different scan rates in ZS


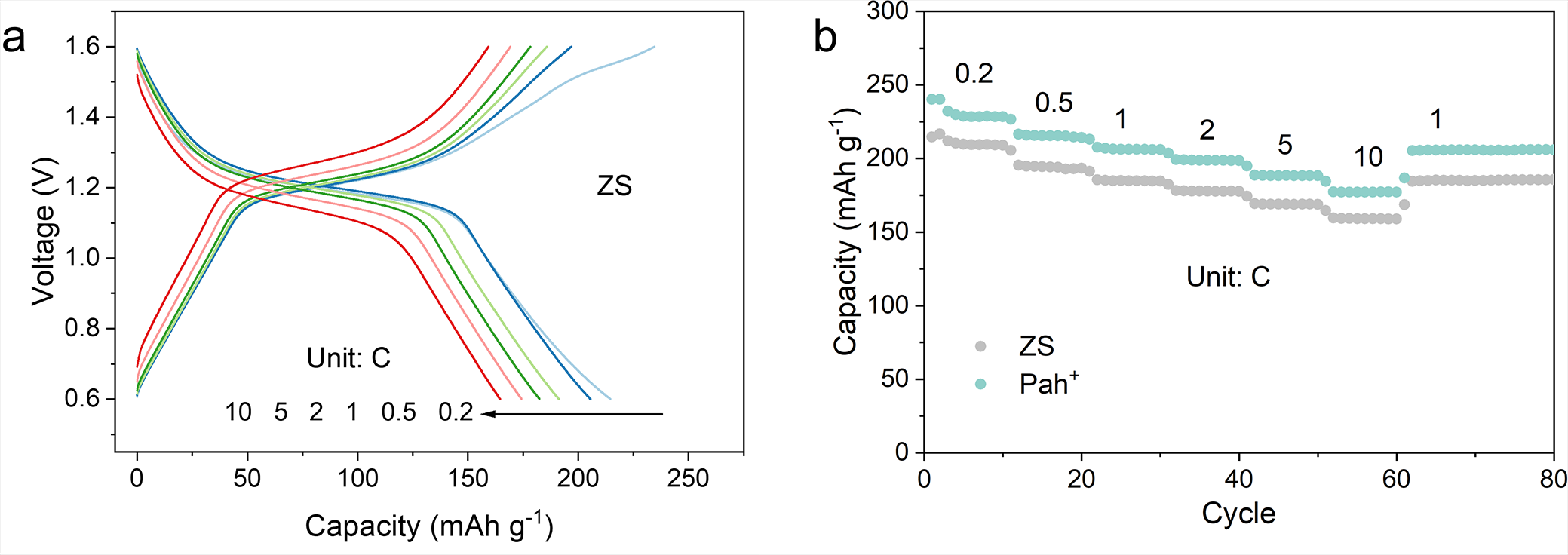


**Fig. S33** Rate capability of Zn-iodine batteries. (**a**) Galvanostatic charge-discharge profiles of Zn-iodine battery in ZS and (**b**) comparison of rate performance in different electrolytes


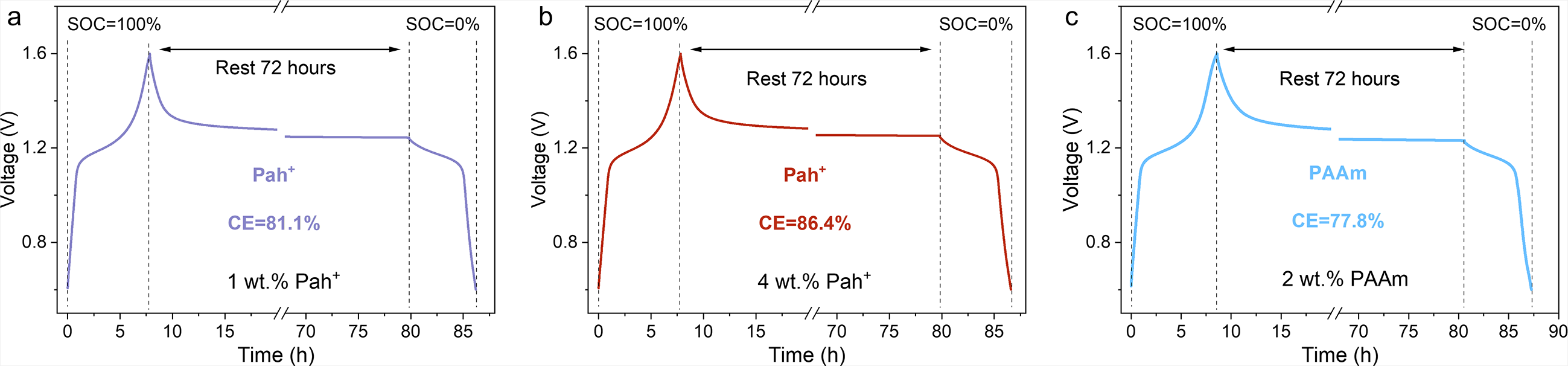


**Fig. S34** Self-discharge behavior of Zn-iodine batteries in 2 M ZnSO_4_ electrolyte with (**a**) 1 wt.% and (**b**) 4 wt.% of Pah^+^, and (**c**) 2 wt.% PAAm

To effectively suppress interfacial side reactions (e.g., corrosion) and stabilize the Zn metal anode, a minimum Pah^+^ concentration of 0.5 wt.% is required. However, this concentration is insufficient to achieve a shuttle-free iodine cathode. Our aging experiments reveal that 2 wt.% Pah^+^ represents the optimal concentration. When using a lower concentration (1 wt.%), the Zn-iodine battery retains 81.1% of its capacity after 72 h, whereas this retention increases to 88.2% at 2 wt.% Pah^+^. Further increasing the concentration to 4 wt.% does not improve performance (86.4% retention), likely due to compromised Zn anode stability from excessive Cl^–^ and sluggish redox kinetics caused by reduced ionic conductivity. Considering both cathode stability and cost-effectiveness, we selected 2 wt.% Pah^+^ as the optimal concentration for this study.

While the neutral containing -NH_2_ groups in the molecular chain, the weak Lewis acid-base interaction and/or ion-dipole interaction between -NH_2_ and polyiodides compared to the Coulombic interactions exhibit limited contribution to polyiodide immobilization. This demonstrates the critical role of cationic sites in long-chain molecules in stabilizing polyiodides and alleviating the shuttle effect.


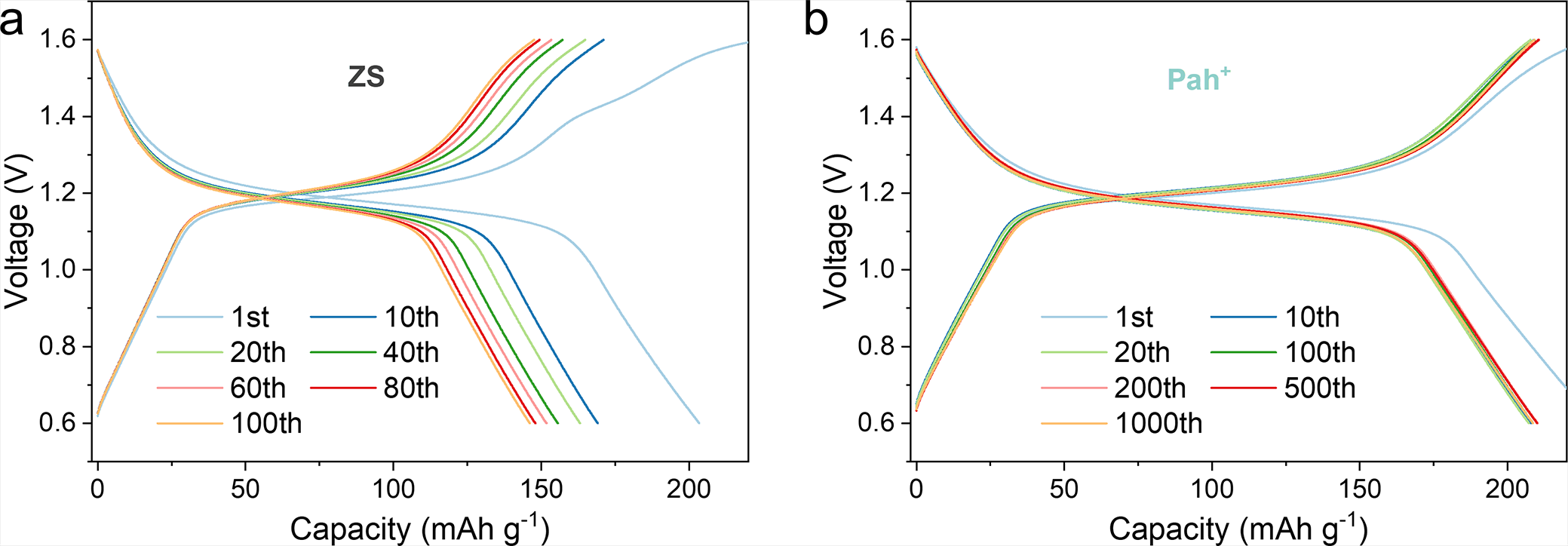


**Fig. S35** GCD curves of Zn-iodine batteries at 1 C in ZS and Pah^+^


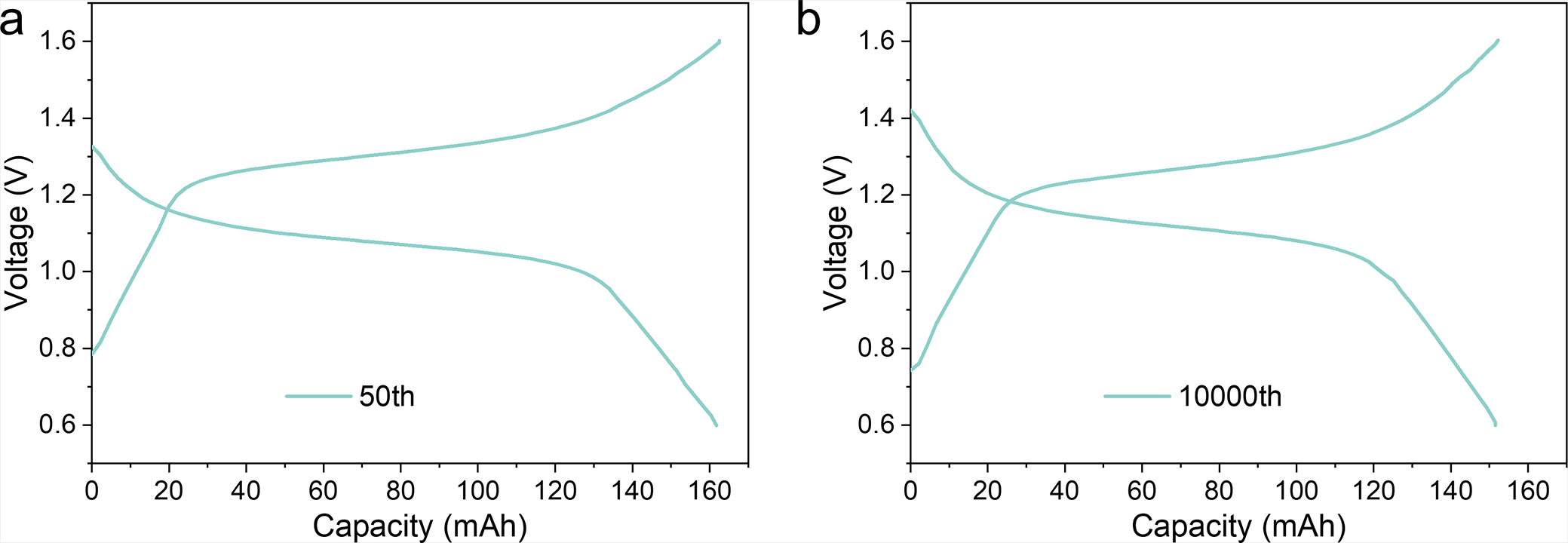


**Fig. S36** GCD curves of Zn-iodine batteries at 10 C in Pah^+^ after (**a**) 50^th^ and (**b**) 10000^th^ cycle


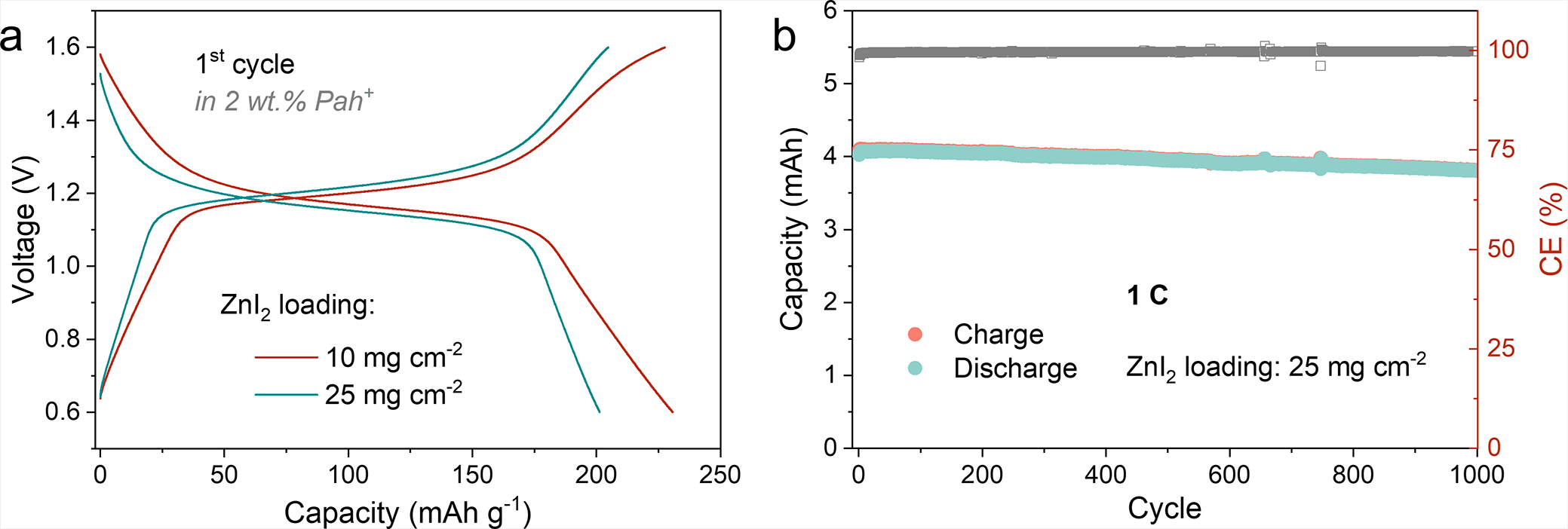


**Fig. S37** (**a**) Comparison of Zn-iodine batteries with different loadings at the first cycle in Pah^+^ and (**b**) the Long-term cycling of Zn-Iodide coin cell at high in Pah^+^ at 1 C


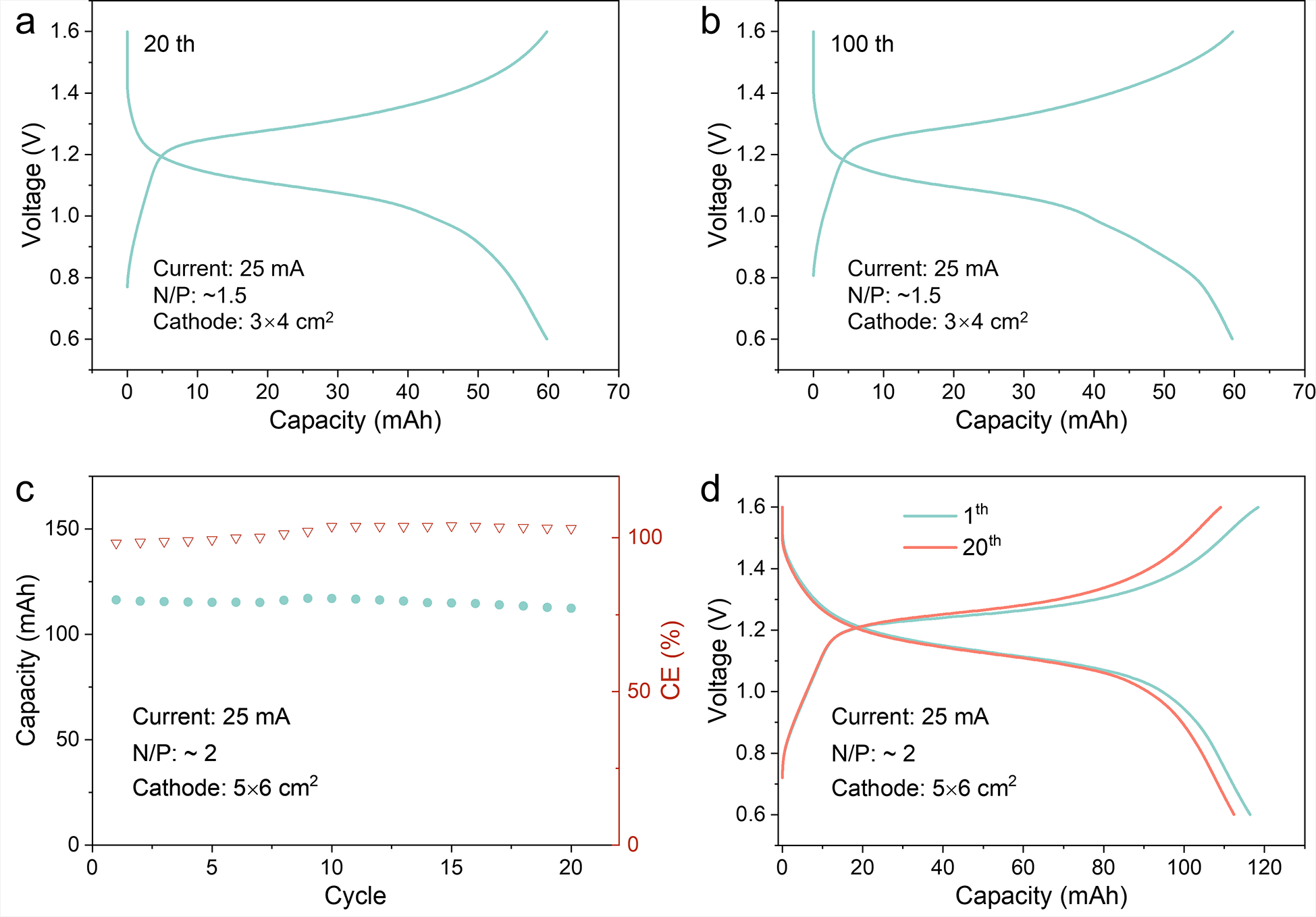


**Fig. S38** (**a**) and (**b**) GCD profiles of the pouch cell with 3×4 cm^2^ cathode at 25 mA, and (**c**) cycling performance and (**d**) GCD curves of Zn-iodine pouch cell with 5×6 cm^2^ cathode


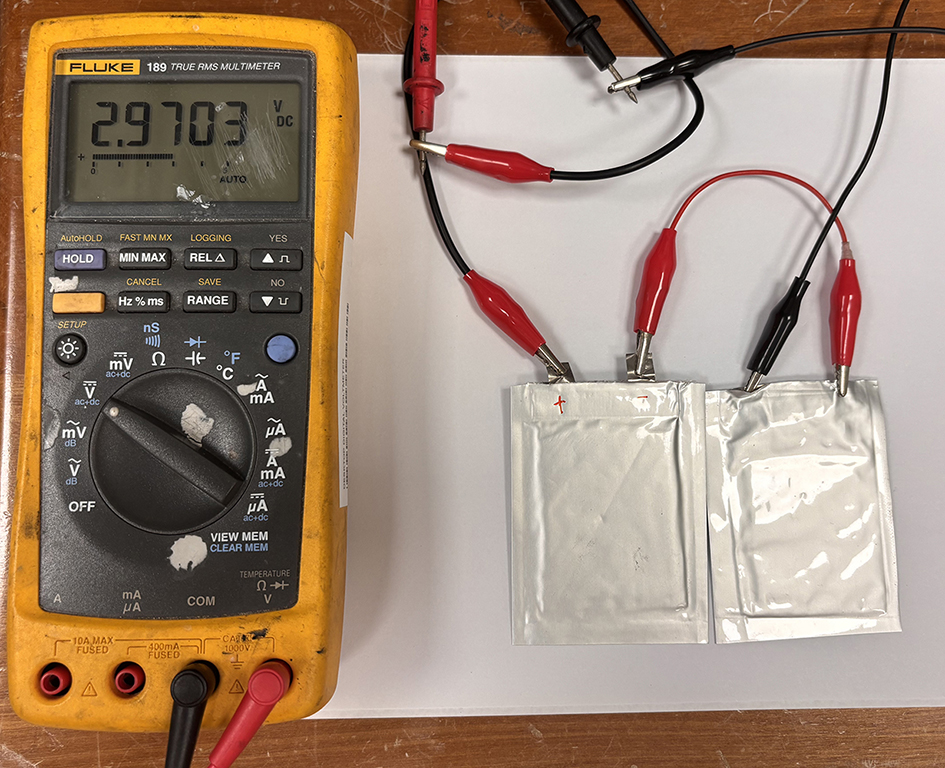


**Fig. S39** Digital photograph of the series circuit of two single-layer pouch cells and the open-circuit potential measured with a multimeter


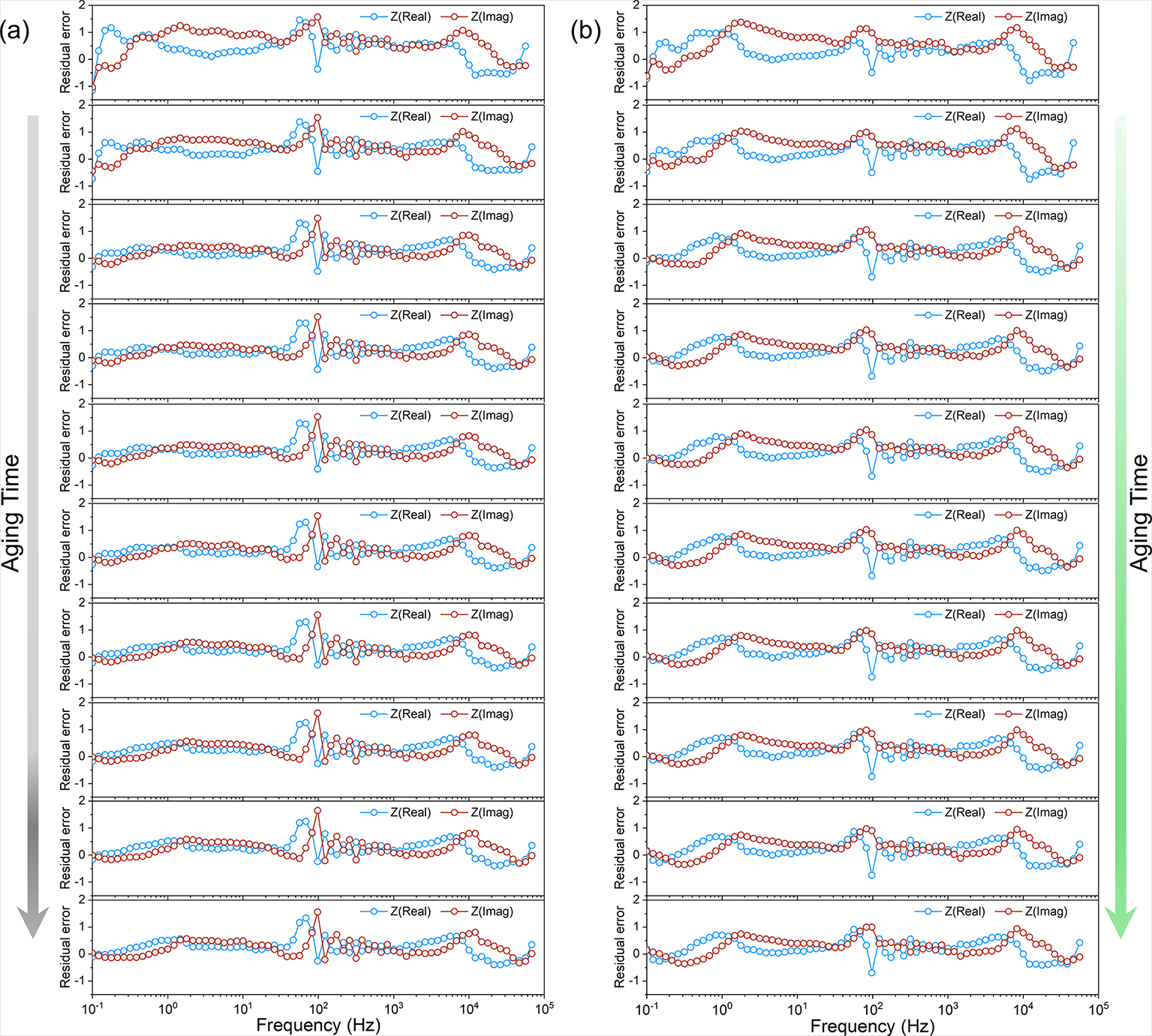


**Fig. S40** Kramers-Kronig validity test of EIS data measured for Zn||Zn symmetric cells in (**a**) ZS and (**b**) Pah^+^ during aging process


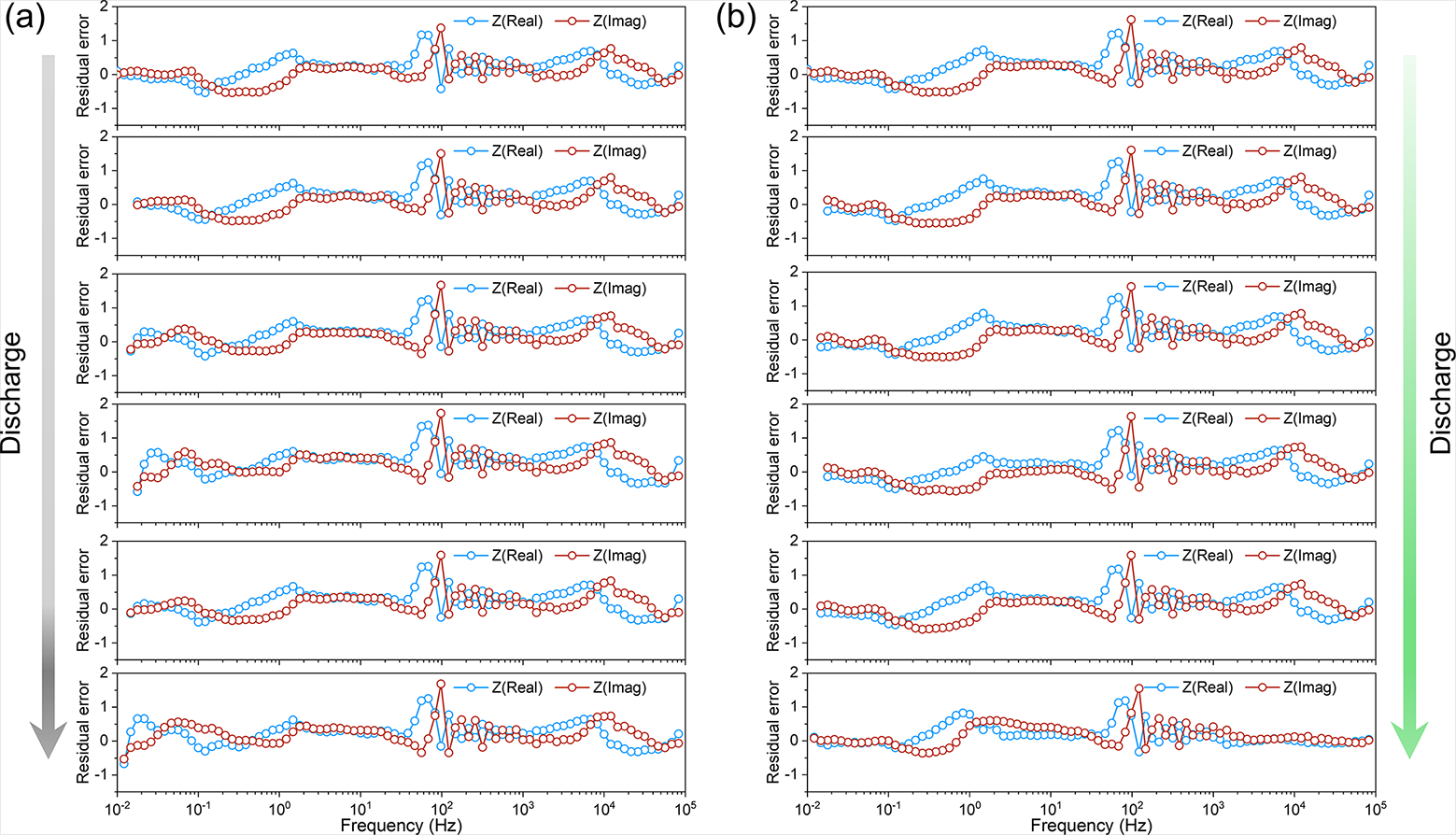


**Fig. S41** Kramers-Kronig validity test of EIS data measured for Zn-iodine batteries in (**a**) ZS and (**b**) Pah^+^ during aging process

**Table S1** Comparison of recently reported performance of Zn metal anodes by additive strategy

| **Baseline electrolyte** | **Additives** | **Testing condition** | **Lifespan** | **Refs.** |
| --- | --- | --- | --- | --- |
| 3 M ZnSO_4_ | 0.05 M Gd^3+^ | 1 mA cm^−2^/1 mAh cm^−2^ | 2100 h | [S2] |
| 2 M ZnSO_4_ | 0.5 M pyridine oxide | 1 mA cm^−2^/1 mAh cm^−2^ | 2300 h | [S3] |
| 1 M ZnSO_4_ | 5-sulfosalicylic acid | 2 mA cm^−2^/0.5 mAh cm^−2^ | 1400 h | [S4] |
| 1 M ZnSO_4_ | D-valine | 2 mA cm^−2^/0.5 mAh cm^−2^ | 4150 h | [S5] |
| 1 M ZnSO_4_ | 75 mM erythritol | 1 mA cm^−2^/1 mAh cm^−2^ | 1800 h | [S6] |
| 2 M ZnSO_4_ | 0.05 M 1-Butyl-3-methylimidazolium Bromide | 1 mA cm^−2^/1 mAh cm^−2^ | 2700 h | [S7] |
| 1 M ZnSO_4_ | 0.5 M 18-crown-6 | 1 mA cm^−2^/1 mAh cm^−2^ | 1700 h | [S8] |
| 1 M Zn(OTf)_2_ | 1 wt.% polysorbate | 1 mA cm^−2^/1 mAh cm^−2^ | 8060 h | [S9] |
| 1 M ZnSO_4_ | 20 mM citric acid+10 mM aspartame | 1 mA cm^−2^/1 mAh cm^−2^ | 4500 h | [S10] |

**Table S2** Comparison of recently reported electrochemical performance of Zn-iodine batteries

| **Strategy** | **Materials** | **I_2_ loading**  **(mg cm^−2^)** | **Capacity**  **(mAh g^−1^)** | **Retention**  **(after cycles)** | **Pouch cell performance** | | **Refs.** |
| --- | --- | --- | --- | --- | --- | --- | --- |
|  |  |  |  |  | **Capacity**  **(mAh cm^−2^)** | **Cycles**  **(Retention)** |  |
| Iodine hosts | Cu-SAC | ~ 2 | 121 (5A g^−1^) | 92.5% (5000) | n.a. | n.a. | [S11] |
|  | Fe-SAC | 1.2 ~ 1.8 | 198.5 (5A g^−1^) | 85.5% (50000) | 3.0 | 200 (82.3%) | [S12] |
|  | B-Fe-NC | ~ 1 | 172 (5 C) | 90% (10000) | ~ 0.6 | 300 (90%) | [S13] |
|  | NS-YP80F@ I_2_ | ~ 1.5 | 127 (50 C) | 76.8% (15000) | ~ 0.2 | n.a. | [S14] |
| Separator  design | UiO-66-(COOH)_2_/GF |  | 164.8 (10 C) | 63% (35000) | n.a. | n.a. | [S15] |
|  | polyPTX | ~ 3.2 | 240 (2A g^−1^) | 96.1% (10000) | ~ 6.4 | 400 (99.6%) | [S16] |
|  | PDDA@GF | 2 ~ 3 | 163 (1 A g^−1^) | 85.8% (10000) | 5.24 | 400 (87.2%) | [S17] |
|  | cCNF/AC@I_2_ |  | 151.3 (3 A g^−1^) | 98.1% (9000) | 2.6 | n.a. | [S18] |
|  | SPES+LAS |  | ~170 (2 C) | 92% (20000) |  |  | [S19] |
| Electrolyte additive | Zinc pyrrolidone carboxylate | ~ 3 mg | 186.7  (10 A g^−1^) | 87% (30000) | n.a | n.a | [S20] |
|  | n-butanol | ~ 1.45 | 0.39 mAh cm^−2^  (5 A g^−1^) | 88.23% (30000) | n.a | n.a | [S21] |
|  | Trimethylamine hydrochloride | ~ 5 | 450 (2 A g^−1^) | 93% (5000) | 1.6 | n.a | [S22] |
|  | 1-(2-hydroxyethyl) imidazole (10 vol%) | 9.7 | 122.2 (2 A g^−1^) | 52.1% (30000) | 1.167 | n.a | [S23] |
|  | Pyridine (~12 mM) | 3.15 | 138.8 (2 A g^−1^) | 92% (10000) | n.a | n.a | [S24] |
|  | Poly(allylamine hydrochloride) | ~ 20 | 230 (1 C) | 91% (1000) | 5.08 | 300 (88.4%) | This work |
|  | Poly(allylamine hydrochloride) | ~ 20 | 170 (10 C) | 93% (10000) | 5.08 | 300 (88.4%) | This work |

# **Supplementary References**

1. M. Yan, C. Xu, Y. Sun, H. Pan, H. Li, Manipulating Zn anode reactions through salt anion involving hydrogen bonding network in aqueous electrolytes with PEO additive. Nano Energy **82**, 105739 (2021). <https://doi.org/10.1016/j.nanoen.2020.105739>
2. L. Jiang, Y. Ding, L. Li, Y. Tang, P. Zhou et al., Cationic adsorption-induced microlevelling effect: a pathway to dendrite-free zinc anodes. **Nano-Micro** Lett. **17**(1), 202 (2025). <https://doi.org/10.1007/s40820-025-01709-0>
3. Z. Li, Z. Wang, W. Sun, Y. Ma, W. Guo et al., Regulating interface engineering by Helmholtz plane reconstructed achieves highly reversible zinc metal anodes. Adv. Mater. **37**(14), 2420489 (2025). <https://doi.org/10.1002/adma.202420489>
4. C. Wang, X. Wang, H. Wang, C. Zheng, S. Tan et al., An organometallic chelated electrolyte based on 5-sulfosalicylic group coordination for elevated performance in aqueous zinc-ion batteries. Adv. Funct. Mater. **n/a**(n/a), 2424024 (2025). <https://doi.org/10.1002/adfm.202424024>
5. J. Lin, C. Ji, G. Guo, Y. Luo, P. Huang et al., Interfacial H-bond network/concentration fields/electric fields regulation achieved by D-valine anions realizes the highly efficient aqueous zinc ion batteries. Angew. Chem. Int. Ed. **64**(24), e202501721 (2025). <https://doi.org/10.1002/anie.202501721>
6. S. Zhang, Q. Gou, W. Chen, H. Luo, R. Yuan et al., Co-regulating solvation structure and hydrogen bond network *via* bio-inspired additive for highly reversible zinc anode. Adv. Sci. **11**(35), e2404968 (2024). <https://doi.org/10.1002/advs.202404968>
7. Y. Lv, C. Huang, M. Zhao, M. Fang, Q. Dong et al., Synergistic anion-cation chemistry enables highly stable Zn metal anodes. J. Am. Chem. Soc. **147**(10), 8523–8533 (2025). <https://doi.org/10.1021/jacs.4c16932>
8. A. Wu, S. Zhang, Q. Li, W. Xue, C. Li et al., Multifunctional crown ether additive regulates desolvation process to achieve highly reversible zinc-metal batteries. Adv. Energy Mater. **15**(19), 2404450 (2025). <https://doi.org/10.1002/aenm.202404450>
9. Z. Peng, S. Li, L. Tang, J. Zheng, L. Tan et al., Water-shielding electric double layer and stable interphase engineering for durable aqueous zinc-ion batteries. Nat. Commun. **16**(1), 4490 (2025). <https://doi.org/10.1038/s41467-025-59830-y>
10. T. Xue, Y. Mu, Z. Zhang, J. Guan, J. Qiu et al., Enhanced zinc deposition and dendrite suppression in aqueous zinc-ion batteries *via* citric acid-aspartame electrolyte additives. Adv. Energy Mater. **n/a**(n/a), 2500674 (2025). <https://doi.org/10.1002/aenm.202500674>
11. F. Yang, J. Long, J.A. Yuwono, H. Fei, Y. Fan et al., Single atom catalysts for triiodide adsorption and fast conversion to boost the performance of aqueous zinc–iodine batteries. Energy Environ. Sci. **16**(10), 4630–4640 (2023). <https://doi.org/10.1039/D3EE01453C>
12. X. Yang, H. Fan, F. Hu, S. Chen, K. Yan et al., Aqueous zinc batteries with ultra-fast redox kinetics and high iodine utilization enabled by iron single atom catalysts. Nano-Micro Lett. **15**(1), 126 (2023). <https://doi.org/10.1007/s40820-023-01093-7>
13. M. Liu, Q. Chen, X. Cao, D. Tan, J. Ma et al., Physicochemical confinement effect enables high-performing zinc-iodine batteries. J. Am. Chem. Soc. **144**(47), 21683–21691 (2022). <https://doi.org/10.1021/jacs.2c09445>
14. W. Feng, Y. Wang, F. Tian, Z. Liu, X. Wei et al., Enhanced carbon host with N-reinforced S-sites to catalyze rapid iodine conversion kinetics for Zn-I2 battery. Energy Storage Mater. **73**, 103812 (2024). <https://doi.org/10.1016/j.ensm.2024.103812>
15. P. Yang, K. Zhang, S. Liu, W. Zhuang, Z. Shao et al., Ionic selective separator design enables long-life zinc–iodine batteries *via* synergistic anode stabilization and polyiodide shuttle suppression. Adv. Funct. Mater. **34**(52), 2410712 (2024). <https://doi.org/10.1002/adfm.202410712>
16. Q. Chen, J. Hao, Y. Zhu, S.-J. Zhang, P. Zuo et al., Anti-swelling microporous membrane for high-capacity and long-life Zn−I2 batteries. Angew. Chem. Int. Ed. **64**(1), e202413703 (2025). <https://doi.org/10.1002/anie.202413703>
17. W. Yuan, X. Qu, Y. Wang, X. Li, X. Ru et al., Polycationic polymer functionalized separator to stabilize aqueous zinc-iodine batteries. Energy Storage Mater. **76**, 104130 (2025). <https://doi.org/10.1016/j.ensm.2025.104130>
18. Z. Li, W. Cao, T. Hu, Y. Hu, R. Zhang et al., Deploying cationic cellulose nanofiber confinement to enable high iodine loadings towards high energy and high-temperature Zn-I_2_ battery. Angew. Chem. Int. Ed **63**(5), e202317652 (2024). <https://doi.org/10.1002/anie.202317652>
19. R. Wang, Y. Liu, Q. Luo, P. Xiong, X. Xie et al., Remolding the interface stability for practical aqueous Zn/I2 batteries *via* sulfonic acid-rich electrolyte and separator design. Adv. Mater. **37**(16), 2419502 (2025). <https://doi.org/10.1002/adma.202419502>
20. F. Wang, W. Liang, X. Liu, T. Yin, Z. Chen et al., A bifunctional electrolyte additive features preferential coordination with iodine toward ultralong-life zinc–iodine batteries. Adv. Energy Mater. **14**(21), 2400110 (2024). <https://doi.org/10.1002/aenm.202400110>
21. W. Yan, Y. Liu, J. Qiu, F. Tan, J. Liang et al., A tripartite synergistic optimization strategy for zinc-iodine batteries. Nat. Commun. **15**(1), 9702 (2024). <https://doi.org/10.1038/s41467-024-53800-6>
22. M. Wang, Y. Meng, M. Sajid, Z. Xie, P. Tong et al., Bidentate coordination structure facilitates high-voltage and high-utilization aqueous Zn−I2 batteries. Angew. Chem. Int. Ed. **63**(39), e202404784 (2024). <https://doi.org/10.1002/anie.202404784>
23. J. Chen, G. Ou, P. Liu, W. Fan, B. Li et al., Pyrrolic-nitrogen chemistry in 1-(2-hydroxyethyl)imidazole electrolyte additives toward a 50, 000-cycle-life aqueous zinc-iodine battery. Angew. Chem. Int. Ed **64**(2), e202414166 (2025). <https://doi.org/10.1002/anie.202414166>
24. Y. Lyu, J.A. Yuwono, P. Wang, Y. Wang, F. Yang et al., Organic pH buffer for dendrite-free and shuttle-free Zn-I2 batteries. Angew. Chem. Int. Ed. **62**(21), e202303011 (2023). <https://doi.org/10.1002/anie.202303011>
